# Contribution of the CR Domain to P-Selectin Lectin Domain Allostery by Regulating the Orientation of the EGF Domain

**DOI:** 10.1371/journal.pone.0118083

**Published:** 2015-02-12

**Authors:** Shouqin Lü, Shenbao Chen, Debin Mao, Yan Zhang, Mian Long

**Affiliations:** 1 Key Laboratory of Microgravity (National Microgravity Laboratory), Institute of Mechanics, Chinese Academy of Sciences, Beijing, China; 2 Center of Biomechanics and Bioengineering, Institute of Mechanics, Chinese Academy of Sciences, Beijing, China; 3 Beijing Key Laboratory of Engineered Construction and Mechanobiology, Institute of Mechanics, Chinese Academy of Sciences, Beijing, China; London, UNITED KINGDOM

## Abstract

The allostery of P-selectin has been studied extensively with a focus on the Lec and EGF domains, whereas the contribution of the CR domain remains unclear. Here, molecular dynamics simulations (MDS) combined with homology modeling were preformed to investigate the impact of the CR domain on P-selectin allostery. The results indicated that the CR domain plays a role in the allosteric dynamics of P-selectin in two ways. First, the CR1 domain tends to stabilize the low affinity of P-selectin during the equilibration processes with the transition inhibition from the S1 to S1’ state by restraining the extension of the bent EGF orientation, or with the relaxation acceleration of the S2 state by promoting the bending of the extended EGF orientation. Second, the existence of CR domain increases intramolecular extension prior to complex separation, increasing the time available for the allosteric shift during forced dissociation with a prolonged bond duration. These findings further our understanding of the structure-function relationship of P-selectin with the enriched micro-structural bases of the CR domain.

## Introduction

The interactions between selectins and their glycoconjugate ligands mediate most adhesions among leukocytes, platelets, and endothelial cells in the response to injury or infection. Typical types of these processes are blood cell tethering to and rolling on endothelial cells to trigger the signaling cascade leading to leukocyte recruitment to sites of inflammation and injury [1,2]. The selectins include three members, P-, E- and L-selectin, which exhibit a common structure consisting of an N-terminal C-type Lectin (Lec) domain, followed by an epidermal growth factor (EGF)-like module, multiple copies of consensus repeats (CRs) (two, six and nine CRs for L-, E- and P-selectin, respectively), a transmembrane segment, and a short cytoplasmic domain [2]. P-selectin glycoprotein ligand-1 (PSGL-1), which is expressed at the top of leukocyte microvilli, plays a key role not only as the ligand of selectins involved in mediating the tethering and rolling of leukocytes but also as the signaling molecule involved in triggering the activation of β_2_ integrin and switching from fast rolling to slow rolling and firm adhesion [3,4]. Under the physiological conditions of blood flow, selectins and ligands are anchored to the opposing surfaces of two cells, their interactions are two dimensional (2D) but not three dimensional (3D) [5], meaning that their interactions are not only determined by their intrinsic association and dissociation kinetics but also are modulated by various extrinsic physical factors, especially the external force resulting from blood flow. The features of the intrinsic rapid forward and reverse rates of all selectin-ligand interactions have been verified to be consistent with their function of modulating the instantaneous capture of blood cells from the blood flow and balancing their rapid rolling [6,7]. The impacts of extrinsic physical factors have also been extensively quantified, such as how the orientation and length of the selectin and the stiffness and microtopology of the molecular carrier affect the on-rate, but not the off-rate of the selectin-PSGL-1 interactions [5,8]. Contact duration and approach velocity affect selectin-PSGL-1 interaction strength [9], probe stiffness regulates both P-selectin-PSGL-1 bond rupture and lifetime [10,11], and extrinsic force could regulate the lifetimes of selectin-ligand interactions [12–14].

The typical features of selectin-ligand interactions are determined by their unique structures. The shallow and dispersive distribution of binding sites located in the Lec domain reveals the structural basis for rapid bond formation and the detachment of selectin-ligand interactions [15]. Despite its remote location with respect to the binding pocket of the Lec domain, the EGF domain plays indispensable roles in modulating selectin-ligand interactions. The concurrence of Lec and EGF (LE) domains constitutes the optimal recognition unit for leukocyte binding [16]. The hinge flexibility between the Lec and EGF domains affects both on-rate and off-rate of L-selectin-ligand interactions [17]. Remodeling of the Lec-EGF domain interface in P- and L-selectin increases adhesiveness and shear resistance under hydrodynamic forces [18]. The impact of Lec-EGF hinge flexibility or remodeling on selectin-ligand interactions indicates the allosteric features of the Lec domain and the regulatory ability of the EGF domain through long distance correlation. Crystallized unligated P-selectin Lec and EGF domains (P-LE) and SGP-3 (a 19 N-terminal sulfoglycopeptide of PSGL-1 composed of three tyrosine sulfate residues, Y605, Y607, and Y610, and an sLe^X^-modified glycan at T616)-ligated P-LE structures exhibit two distinct conformations of P-LE domains [15], which further suggest the allosteric potential of the P-LE domains. A allosteric model is proposed based on the crystallized structures, which predicts that tensile force exerted on a selectin-ligand complex could induce extension of the EGF orientation and further trigger the allostery of P-selectin from the low-affinity to the high-affinity conformation through transmission from the EGF-Lec domain interface to the ligand-binding interface in the lectin domain [19]. The allosteric dynamics and the relationship between the binding pocket conformation of the P-selectin Lec domain and the orientation of the EGF domain have been investigated using molecular dynamics (MD) simulations [20], and the results predict the existence of a new conformation of the Lec domain that is distinct from both crystallized conformations.

The functions of Lec and EGF domains of selectins have mainly been addressed from biophysical and microstructural perspective because of their direct and distinct contributions to selectin-ligand interactions. While the understanding of CR domain function is limited, the presence of CR domains is known to enhance the intrinsic on-rate of the P-selectin-PSGL-1 interaction *via* conferring a sufficient length and flexibility of P-selectin [5], in addition to increasing the elasticity of selectins [21]. However, the question remains that whether the CR domains contribute to the microstructural features of selectins, and if so, how do the CR domains affect conformational dynamics and selectin-ligand interactions? Thus, we investigated the impacts of CR domains on the conformational dynamics of P-selectin during both free equilibration and forced dissociation through the combination of homology modeling and molecular dynamics (MD) simulations.

## Methods

### Construction of the simulation system

**Fig 1:**
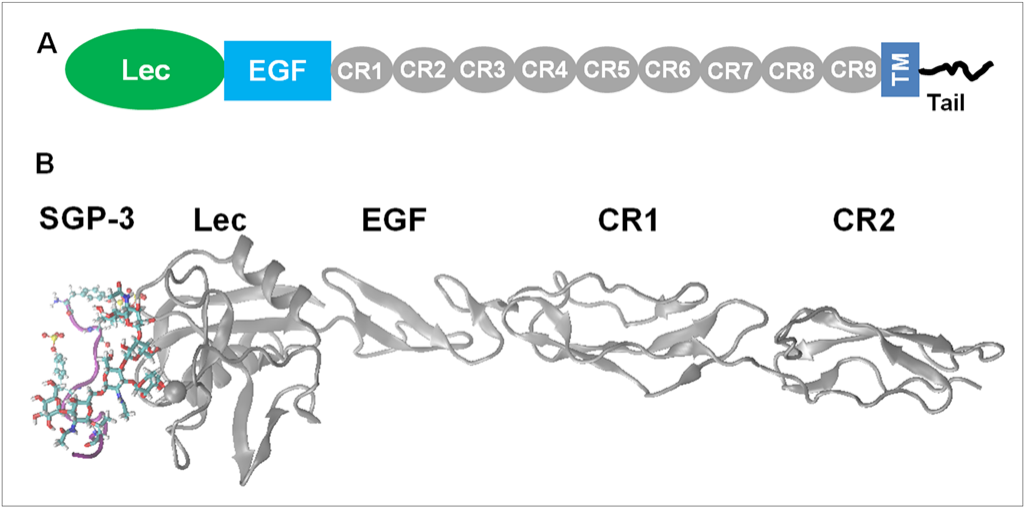
P-selectin components and simulation system. (*A*) Schematic representation of P-selectin domain components. (*B*) Maximal components of the P-selectin-PSGL-1 complex for the simulations, which included the Lec, EGF, CR1 and CR2 domains of P-selectin and the SGP-3 ligand of PSGL-1.

Based on the components of P-selectin (Fig. 1*A*), crystallized unligated P-LE domains (PDB code: 1G1Q) and SGP-3-ligated P-LE domains (PDB code: 1G1S) as well as homology-based models of the first two CR domains of P-selectin were employed to build simulation systems (Fig. 1*B*). The strontium ion was replaced with a calcium ion in all of the simulation systems that included SGP-3-ligated P-LE domains. The first two CR domains of P-selectin were modeled using the homology model module in Molecular Operating Environment (MOE) based on six templates (PDB codes: 2G7I, 1RID, 1OK3, 1GKN, 2RLQ, 1PPQ), and the selection of templates was based on a combination of MOE (E-value ≤ 10^–12^) and Blast (Blast-score ≥ 25) sequence homology searches [22]. The modeled structures based on the 2RLQ template were chosen in this study by considering stereochemistry structure optimization (S1 Table) as well as the similarity of the disulfide bond distribution between P-selectin CR domains and the template (S2 Table). After further optimization of 10 nanosecond (*ns*) MD equilibration simulations, the resulting CR domains were used to construct simulation system. Three sets of simulation systems were built in this study for testing the impact of CR domains on P-selectin function. *Set I*, or 0CR, without a CR domain, included two unligated P-LE systems separated from 1G1Q (designated 1G1Q/P-LE) and from 1G1S (designated 1G1S/P-LE), while their corresponding SGP-3-ligated complexes were designated 1G1Q/P-LE-SGP-3 and 1G1S/P-LE-SGP-3, respectively. *Set II*, or 1CR, with the first CR domain, also included four systems that corresponded to *Set I* by adding the first CR domain to every system. The orientation of the CR domain to the EGF domain was determined through sequence alignment between P-selectin and the 2RLQ template and followed structural superposition. Firstly, the sequence alignment of both EGF domain and CR1 domain with that of 2RLQ was performed, then the structures of P-LE and modeled CR1 domain were superposed to x-ray crystal 2RLQ, respectively, based on the backbone alignment of corresponding residues in aligned sequence, and finally the C-term of EGF domain and N-term of CR1 domain were covalently linked. *Set III*, or 2CR, with first two CR domains, was only composed of two complexes, which corresponded to *Set II* but included one more CR domain, and the orientation of the second CR domain to the first CR domain was determined using a similar method to *Set II* (Table 1). Each simulation system was built by solvating the target molecule into a rectangular water box, which was then neutralized by adding 100 mM sodium and chlorine ions to mimic the physiological ionic concentration.

**Table 1:** Summary of the simulation set-up.

Set	System	Free MD (Duration (ns) × Runs)	Force-induced dissociation	
			SMD set	Mode × Runs
0CR	1G1Q/P-LE	45×1; 30×5		
	1G1S/P-LE	30×5; 60×1		
	1G1Q/P-LE-SGP3	30×2		
	1G1S/P-LE-SGP3	25×1; 30×1		
1CR	1G1Q/P-LEC	20×1; 30×5		
	1G1S/P-LEC	20×1; 60×1;30×4		
	1G1Q/P-LEC-SGP3	20×1; 30×1	EGF end fixed	*cf*_300 × 6
			CR1 end fixed	*cf*_300 × 6
	1G1S/P-LEC-SGP3	20×1; 30×1		
2CR	1G1Q/P-LECC-SGP3	20×1; 30×1	EGF end fixed	*cf*_300 × 6
				*cv*_0.01 × 6
			CR1 end fixed	*cf*_300 × 6
				*cv*_0.01 × 6
			CR2 end fixed	*cf*_300 × 6
				*cv*_0.01 × 6
	1G1S/P-LECC-SGP3	20×1; 30×1	EGF end fixed	*cf*_300 × 6
				*cv*_0.01 × 6
			CR1 end fixed	*cf*_300 × 6
				*cv*_0.01 × 6
			CR2 end fixed	*cf*_300 × 6
				*cv*_0.01 × 6

1G1Q/P-LE: the P-selectin system including only the Lec and EGF domains, which were separated from crystallized 1G1Q;

1G1Q/P-LE-SGP3: the corresponding SGP-3-ligated 1G1Q/P-LE system;

1G1Q/P-LEC: the P-selectin system including the Lec, EGF and CR1 domains, which was built by docking the CR1 domain with the P-LE domains of 1G1Q;

1G1Q/P-LEC-SGP-3: the corresponding SGP-3-ligated 1G1Q/P-LEC system;

1G1Q/P-LECC-SGP3: the SGP-3-ligated P-selectin system including the P-selectin Lec, EGF, CR1 and CR2 domains, which was built by docking the first two CR domains with the P-LE domains of 1G1Q.

Similar names were used for the 1G1S/P-LE, 1G1S/P-LE-SGP-3, 1G1S/P-LEC, 1G1S/P-LEC-SGP-3 and 1G1S/P-LECC-SGP-3 systems.

### Equilibration and forced dissociation simulations

Two types of MD simulations were conducted in this study: equilibration and forced dissociation simulations. Prior to the equilibration process, energy minimization was initiated with 10,000 steps by fixing protein backbone atoms or sugar heavy atoms, followed by an additional 10,000 steps with all atoms free. System heating was then performed from 0 to 300 *K* at 30 *K* increments every 5 picoseconds (*ps*). Two and six independent runs involving relaxation of no less than 20 *ns* in each run were conducted for SGP-3 ligated and SGP-3 ligand free systems, respectively. Based on the final snapshots of the first equilibration run, forced dissociation simulations were executed for the 1G1Q/P-LEC-SGP-3 complex of *Set II* and both complexes of *Set III* under a constant force (*cf*) of 300 piconewtons (*pN*) or a constant velocity (*cv*) of 0.01 Å/*ps*, with different fixed ends (Table 1). The C-terminal P618-C_α_ atom of SGP-3 ligand peptide was pulled along the vector from the fixed end to the pulled end in all dissociation simulations, and a spring constant of 70 *pN*/Å was set in all *cv-*0.01 Å/*ps* dissociation simulations. Six repeats were carried out for each dissociation setting. The NAMD program and its steered molecular dynamics (SMD) module were employed for equilibration and forced dissociation simulations, respectively, using a CHARMM27 all-atom force field for proteins and a self-built force field for six sugar residues and three tyrosine sulfate residues in the SGP-3 ligand [23–25]. An integration time step of 1 femtosecond (*fs*) and periodic boundary conditions were applied in the simulations. A smooth (10–12 Å) cutoff and the Particle Mesh Ewald (PME) method were employed to calculate van der Waals forces and full electrostatics, respectively. The 300 *K* heat bath was manipulated using a Langevin thermostat, and the 1 *atm* pressure was controlled through the Nosé-Hoover Langevin piston method in the equilibration processes.

### Data analyses

Three aspects were emphasized in the simulation analyses. First, the conformational dynamics during both the equilibration and forced dissociation processes were evaluated according to the root of mean standard deviation (RMSD), the orientations of the EGF domain and the first CR (CR1) domain. The RMSDs of the *R*3 loop (P81-D89) relative to different references were calculated to investigate the allostery of the Lec domain based on the alignment of rigid parts of the Lec domain. The EGF orientation was characterized by the relative angle of the EGF domain in the simulation systems to crystallized 1G1Q or 1G1S based on the alignment of the rigid parts of the Lec domain, which was quantified using the two vectors connecting the geometric center of the heavy atoms of the Lec-EGF hinge (residues A120 and S121) of crystallized 1G1Q or 1G1S to that of main EGF domain (residues C122 to T141) of crystallized 1G1Q or 1G1S and the simulation trajectories (S1 Fig., *A*). The CR1 domain orientation was characterized by the relative angle of the CR1 domain in the simulation trajectories to the corresponding initial system, which was indicated by the angle between the two vectors connecting the geometric center of the heavy atoms of the EGF-CR1 hinge (residues C159 and G160) of the initial conformation to that of the CR1 domain (residues E161 to L217) of the initial system and the equilibrated trajectories, based on the alignment of the EGF domain (S1 Fig., *B*). Second, the strength of the nonbond interaction between P-selectin and PSGL-1 or between different P-selectin domains was evaluated for both the equilibration and dissociation processes, including van der Waals and electrostatic interactions. The nonbond interactions reaching zero was set as the criterion for calculating the dissociation time of the P-selectin-PSGL-1 interaction in the *cv-*0.01 Å/*ps* or *cf-*300 *pN* forced unbinding simulations. Lastly, the dynamics of the forced dissociation processes were described based on the force profiles, dissociation times, molecular extensions and complex separations. The molecular extension was defined according to the displacement of the pulled P618-C_α_ atom of the SGP-3 ligand peptide along the force vector, and complex separation was quantified based on either the distance between the geometric center of the P-selectin Lec domain and that of the SGP-3 ligand along the force vector, or the distance between the geometric center of the fucose (Fuc) of the SGP-3 sugar and calcium ion. The system construction and data analyses were performed using the VMD program [26].

## Results

### Homology modeling of the first two CR domains of P-selectin

Determining the structure of the CR domain of P-selectin is a requirement for investigating the impact of the CR domain using MD simulations. Based on sequence homology searches by both MOE and Blast, six templates (PDB codes: 2G7I, 1RID, 1OK3, 1GKN, 2RLQ, 1PPQ) were chosen for the homology modeling of P-selectin CR domains. However, only the modeled structures based on the 2RLQ and 1OK3 templates showed low ratios of unreasonable structures (S1 Table, *gray boxes*) according to stereochemistry rationality, and the disulfide bond distributions in templates 2G7I and 2RLQ (*B*) were similar with that found in the P-selectin CR domain (*A*) (S2 Table). Furthermore, the 2RLQ template exhibited the highest sequence homology with the CR domain (S1 Table, *second column*) as well as with the C terminus of the EGF domain, which helped us to build a simulation system with reasonable settings regarding the CR domain orientation with respect to the EGF domain by combining sequence alignment and structure superposition between the P-selectin target and the template. Integrated with the above three considerations, the structures of the P-selectin CR domains modeled based on the 2RLQ template were adopted in this study. In fact, comparisons among all of the modeled structures based on the six templates demonstrated conformational similarity, with small, stable backbone C_α_ distances for each residue, except for the first twenty residues of the N terminus (S2 Fig., *A*). The large fluctuation of the N terminal conformation resulted from the lack of N terminal disulfide bonds 1–5 in some templates (S2 Table, *B*). The compact, ellipsoid structure, with a length of approximately 3.8 *nm*, observed for the modeled CR domain (S2 Fig., *B*) was consistent with the characteristics observed under electron microscopy [27] and from prediction [28]. Furthermore, the modeled structure of P-selectin first CR domain was similar with that of newly X-ray crystallized E-selectin first CR domain with C_α_ atom RMSD of ~2.0 Å (S2 Fig., *C*), where their conformational difference was comparable with that between the first and the second CR domains of E-selectin (S2 Fig., *D*), and the modeled CR1 orientation relative to EGF domain of P-selectin was also consistent with that of E-selectin (S2 Fig., *E*). The structural reliability of the CR domain laid the foundation for further investigating its impact on the conformation of P-selectin.

### Allosteric features of P-selectin

**Fig 2:**
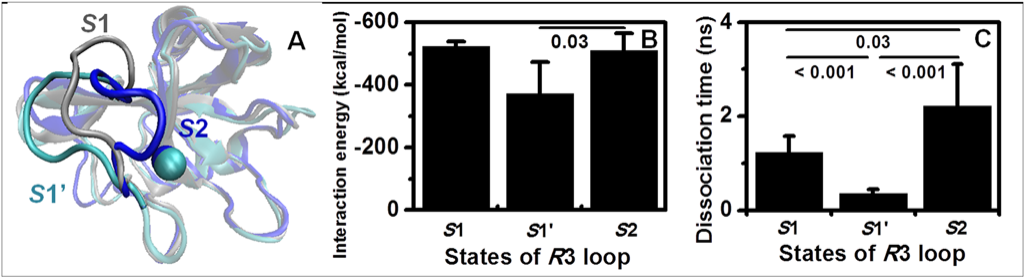
Features of P-selectin with different conformations of the *R*3 loop. (*A*) Conformational differences in the *R*3 loop between *S*1 (*silver*), *S*1’ (*cyan*) and *S*2 (*blue*). The Lec domain is presented in *newcartoon* format in *topview*; the other components, except for the *R*3 loop and calcium ion, are shown in *transparent* format for clarity. The *S*1’ conformation is the final snapshot of the 20-*ns* equilibration of the 1G1Q/P-LEC system of the 1CR set. (*B*) Differences in receptor-ligand interactions for P-selectin with distinct *R*3 conformations of *S*1, *S*1’ and *S*2 during the free equilibration process of SGP-3-ligated systems in the 0CR, 1CR and 2CR sets. Non-covalent energy, including van der Waals and electrostatic interactions between P-selectin and the SGP-3 ligand, were quantified, and only the snapshots showing an *R*3 loop RMSD of less than or equal to 4.0 Å in relation to the reference state *S*1, *S*1’ or *S*2 were counted. The trajectory was counted in statistical analyses of state *S*1’ if its snapshots fit the rules of both *S*1 and *S*1’. The data are presented as the mean ± SD of the different equilibration systems. (*C*) Differences in dissociation time between the complexes with distinct *R*3 loop conformations of *S*1, *S*1’ and *S*2 under a constant force of 300 *pN* pulling on the SGP-3 ligand with EGF end fixation. The data were presented as the mean ± SD of six dissociation runs for each system. The dissociation simulations were based on the final state after a 20 *ns* equilibration of the 1G1Q/P-LECC-SGP3, 1G1Q/P-LEC-SGP3, and 1G1S/P-LECC-SGP-3 systems for *S*1, *S*1’ and *S*2, respectively.

The crystallized unligated P-LE domains present two features that are distinct from the SGP-3-ligated P-LE domains [15]. The former structure presents bent EGF domain (S3 Fig., *A*, *sliver*) and flat lying *R*3 loop (S3 Fig., *B*, designated *S*1 in *sliver*) within the plane of the binding pocket, and the latter structure presents extended EGF orientation (S3 Fig., *A*, *blue*) with upright *R*3 loop (S3 Fig., *B*, designated *S*2 in *blue*) [19,20]). Free equilibration simulations of the two distinct P-LE systems did not reveal the direct allostery between *S*1 and *S*2. A new stable conformation of the *R*3 loop labeled as *S*1’ (Fig. 2*A*, *silver*), with typical features of anticlockwise rotation from the *S*1 state and reverse verticality from the binding pocket plane (S3 Fig., *E*, *cyan*), appeared during the relaxation process of the 1G1Q/P-LE system in the 0CR set (S3 Fig., *C*) [20]. This conformation could rise up slightly to easily allow ligand binding (S3 Fig., *G*, *cyan*), similar to the clockwise relaxed conformation from the *S*2 state (S3 Fig., *F*-*G*, *purple*) observed during the equilibration of the 1G1S/P-LE system of the 0CR set (S3 Fig., *D*).

This conformation of *S*1’ appeared frequently during the equilibration evolution of the 1G1Q systems for the 0CR and 1CR sets, and its distinction from both the *S*1 and *S*2 states was further confirmed in the following evaluations. First, the different states of the Lec domain *R*3 loop resulted in distinct P-selectin-PSGL-1 interactions, where the unfavorable conformation of the *S*1’ state showed the weaker non-covalent interaction energy (*middle*) compared with those for the *S*1 (*left*) and *S*2 (*right*) states (Fig. 2*B*). It is necessary to note that SGP-3-ligated P-selectin would constrain the freedom of the *R*3 loop and reduce the allosteric shift of *S*1 to *S*1’ [20], leading the remarkably decreased occurrence and extent of allostery from *S*1 to *S*1’ for the SGP-3-ligated systems compared with the noligated systems. Therefore, the sample numbers of the equilibration systems were lower for the statistics related to the *S*1 (3 runs) and *S*1’ (3 runs) states than for the *S*2 (6 runs) state, and there was therefore no significant difference in interaction energy between *S*1 and *S*1’, even though the former showed slightly stronger interactions and a lower standard deviation compared with the *S*2 state (Fig. 2*B*). Second, receptor-ligand interactions determine dissociation behavior. Based on the final equilibrated states of the SGP-3-ligated complex systems with different conformations of *S*1 (1G1Q/P-LECC-SGP-3), *S*1’ (1G1Q/P-LEC-SGP-3) and *S*2 (1G1S/P-LECC-SGP-3), six independent runs of forced dissociation under a constant force of 300 *pN* for each system were carried out with D158-C_α_ fixation of the EGF domain C-terminus. The bond dissociation time of *S*1’ decreased significantly compared with those of *S*1 and *S*2 states (Fig. 2*C*). The comparisons of SGP-3 ligand interaction energy of equilibration simulations and the bond dissociation time of forced unbinding among different *R*3 loop states further confirmed the existence of the intermediate state, *S*1’, of the Lec domain, which potentially bridges the allostery between *S*1 and *S*2 [20].

### Impact of the CR domain on P-selectin allostery during the equilibration processes

**Fig 3:**
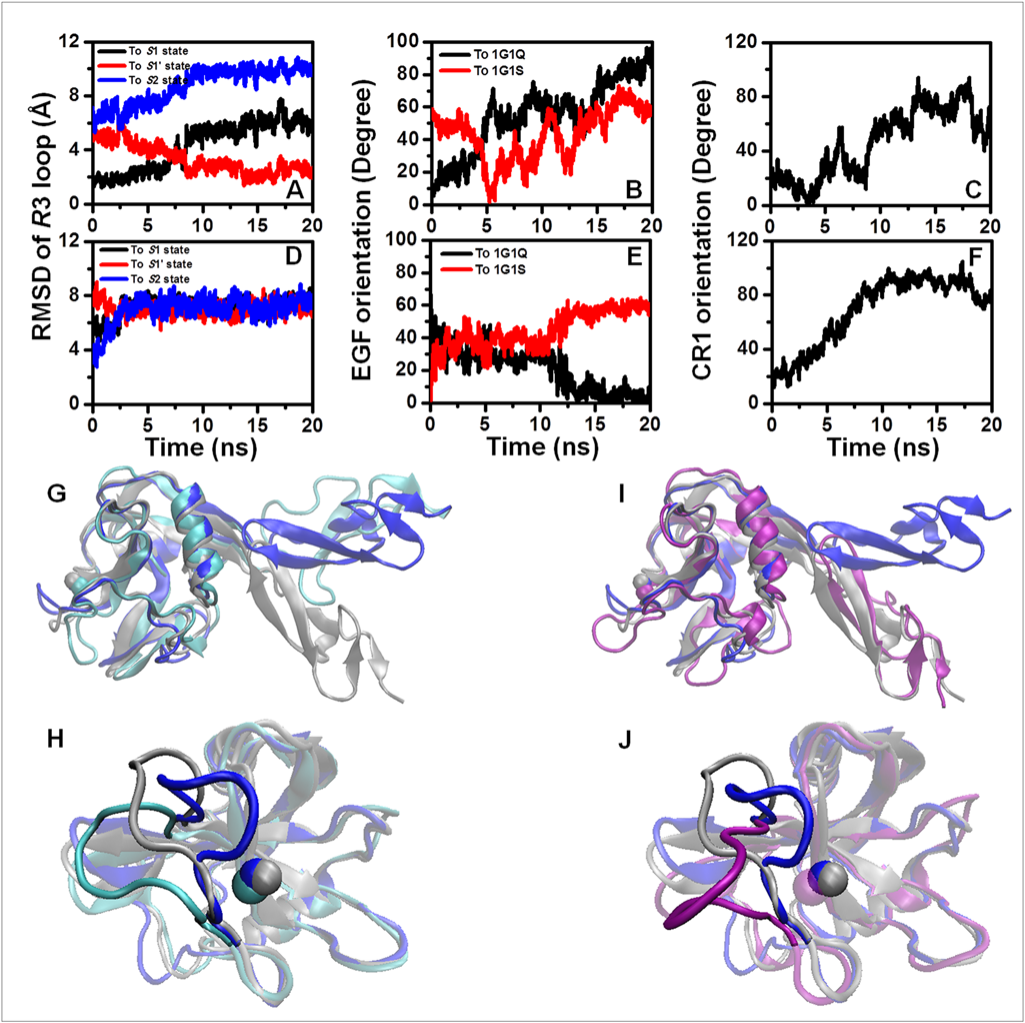
Typical features of the equilibration processes for the 1G1Q/P-LEC and 1G1S/P-LEC systems. (*A*, *D*) The conformation of the Lec domain quantified according to the RMSDs of the *R*3 loop with respect to the references *S*1 (*black*), *S*1’ (*red*) and *S*2 (*blue*) based on the alignment of the rigid regions of the Lec domain. (*B*, *E*) The EGF domain orientation with respect to the references crystallized 1G1Q (*black*) and 1G1S (*red*). (*C*, *F*) The orientation of the CR1 domain relative to the initial conformation. The 20-*ns* equilibration process for the unligated 1G1Q/P-LEC (*A*-*C*) and 1G1S/P-LEC (*D*-*F*) systems of the 1CR set is featured here, and the corresponding final snapshots (*cyan*, *purple*) developed from 1G1Q (*G*, *H*) and 1G1S (*J*, *I*) are illustrated to highlight the EGF orientations (*G*, *J*) and *R*3 loop (*H*, *I*). The crystallized references 1G1Q (*silver*) and 1G1S (*blue*) are superposed for comparison.

To investigate the contribution of CR domain, we compared the dynamic differences in the equilibration processes between the systems without the CR domain and those with one or two CR domains (Table 1). Overall, the unligated systems of the 1CR set showed similar conformational regulations with those of the 0CR set (Fig. 3, S4–S7 Figs.). A typical equilibration of the 1G1Q/P-LEC system demonstrated the shift of the Lec domain from initial *S*1 state to *S*1’ state after gradual evolution over 2.5–8.0 *ns* (Fig. 3*A*). Correspondingly, the EGF domain experienced a transition from the initial bent orientation to an extended orientation, with short-lived consistency, to crystallized 1G1S at ~ 5 *ns*, followed by hovering during 5–12.5 *ns* and further departure (*red*) (Fig. 3*B*). It was not difficult to determine that the transitions of the EGF orientation took place earlier than those of the *R*3 loop conformation of the Lec domain (Fig. 3*A*-*B*). However, the orientation of the first CR domain exhibited much earlier deviation from its initial position than that of the EGF domain (Fig. 3*C*). The final snapshot of the 20-*ns* equilibration intuitively revealed the adjusted EGF orientation (Fig. 3*G*, *cyan*) and the adopted conformation of the Lec domain *R*3 loop (Fig. 3*H*, *cyan*). The forward and backward order of the time-dependent evolution for the transition of the CR1 domain orientation, EGF domain orientation and Lec domain conformation could be observed intuitively during the dynamic process of 20-*ns* equilibration (S1 Movie).

A typical equilibration of the 1G1S/P-LEC system revealed abrupt and quick relaxation of the *R*3 loop from the *S*2 state (Fig. 3*D*), and the EGF orientation deviated from the crystallized extended orientation to the crystallized bent orientation (Fig. 3*E*). The orientation of the CR1 domain displayed continuous movement away from initial position in beginning until ~9 *ns* (Fig. 3*F*). A visual snapshot of the final state of the simulation was obtained by focusing on the EGF orientation (Fig. 3*I*, *purple*) and the *R*3 loop conformation of the Lec domain (Fig. 3*J*, *purple*). Again, the forward and backward order of the time-dependent evolution for the transitions of the CR1 domain orientation, EGF domain orientation and Lec domain conformation could be observed intuitively during the dynamic process of 20-*ns* equilibration (S2 Movie). The observed order in both typical equilibrations of 1G1Q/P-LEC and 1G1S/P-LEC systems confirmed the ability of the EGF domain to regulate the conformation of the Lec domain by mediating its orientation and long distance correlation between EGF and Lectin domains and suggested that the CR1 domain functioned in regulating the conformation of the Lec domain by mediating the orientation of the EGF domain.

**Fig 4:**
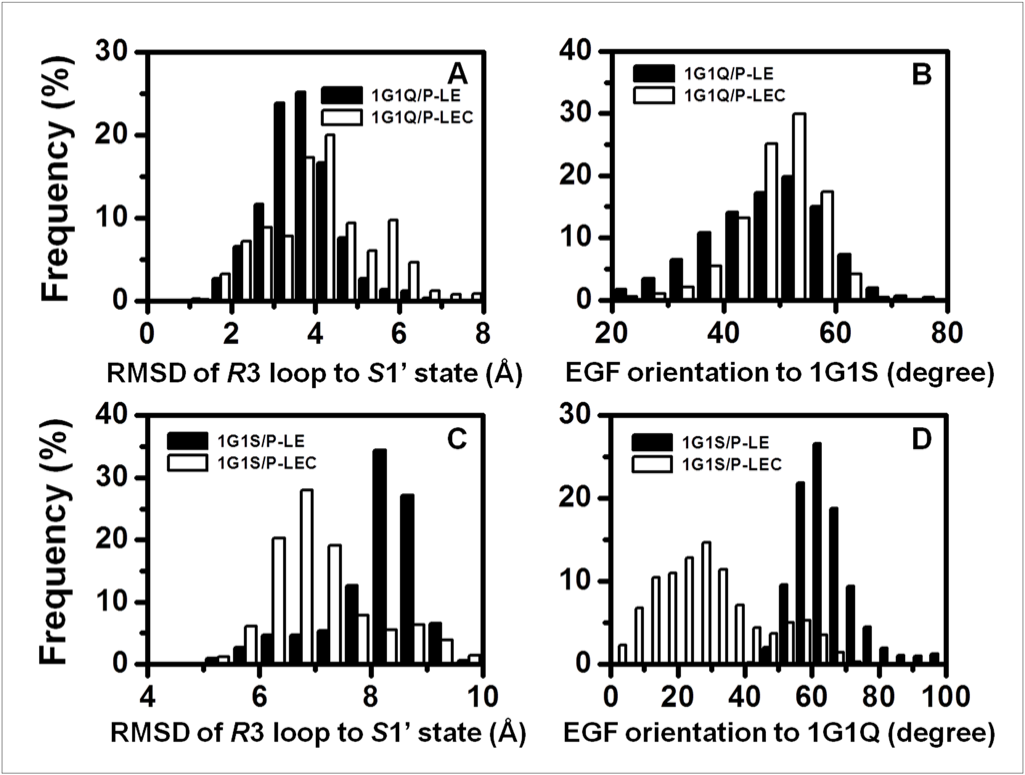
Impact of the CR domain on the equilibration process. Differences of the conformational features of Lec domain *R*3 loop (*A*, *C*) and the EGF orientation (*B*, *D*) between equilibration processes of non-ligated 0CR (*black*) and 1CR (*white*) sets of 1G1Q (*A*, *B*) and 1G1S (*C*, *D*) systems. The conformational features of Lec domain *R*3 loop was quantified by the RMSD for the *S*1’ state based on alignment of the rigid parts of the Lec domain, and the EGF orientation was calculated based on the references of crystallized 1G1S and 1G1Q for the 1G1Q and 1G1S systems, respectively. The data were from six equilibration processes for each system.

We further compared the differences between non-ligated 1G1Q/P-LE (S4 Fig.) and 1G1Q/P-LEC (S5 Fig.) systems as well as between non-ligated 1G1S/P-LE (S6 Fig.) and 1G1S/P-LEC (S7 Fig.) systems during equilibration processes. The results demonstrated that the CR1 domain inhibited the extending of the EGF domain from the initial bent orientation (Fig. 4*B*) and corresponding shift of Lec domain *R*3 loop from initial *S*1 state to *S*1’ state (Fig. 4*A*) in the 1G1Q systems, and that the CR1 domain promoted the bending of the EGF domain from the initial extend orientation (Fig. 4*D*) and corresponding relaxation of Lec domain *R*3 loop (Fig. 4*C*). Even the extent of CR1 contribution to P-LE conformational stability was weaker for 1G1Q systems than that for 1G1S systems, the features that the CR1 domain tends to regulate the EGF domain to bent orientation and the Lec domain *R*3 loop to *S*1 state were consistent. The results indicated that the existence of the CR domain not only provided physical support to allow sufficient extension of the length of P-selectin for binding to its counterpart but also played an important role in the regulation of P-selectin conformational dynamics and, further, P-selectin-PSGL-1 interaction by conformational propagation of the EGF reorientation to Lectin domain based on the long distance correlation between Lectin and EGF domains.

### Impact of the CR domain on the cv forced dissociation processes of P-selectin-PSGL-1 complex

Forced dissociation is a typical feature of P-selectin-PSGL-1 interactions to mediate the rolling adhesion of leukocytes under blood flow. To investigate the contribution of the CR domain to the forced dissociation process, we performed SMD simulations under *cv*-0.01 Å/*ps* and *cf*-300 *pN* based on the SGP-3-ligated systems of the 2CR set as well as the SGP-3-ligated 1G1Q system of the 1CR set with different C terminal fixations of the EGF, CR1 or CR2 domain.

**Fig 5:**
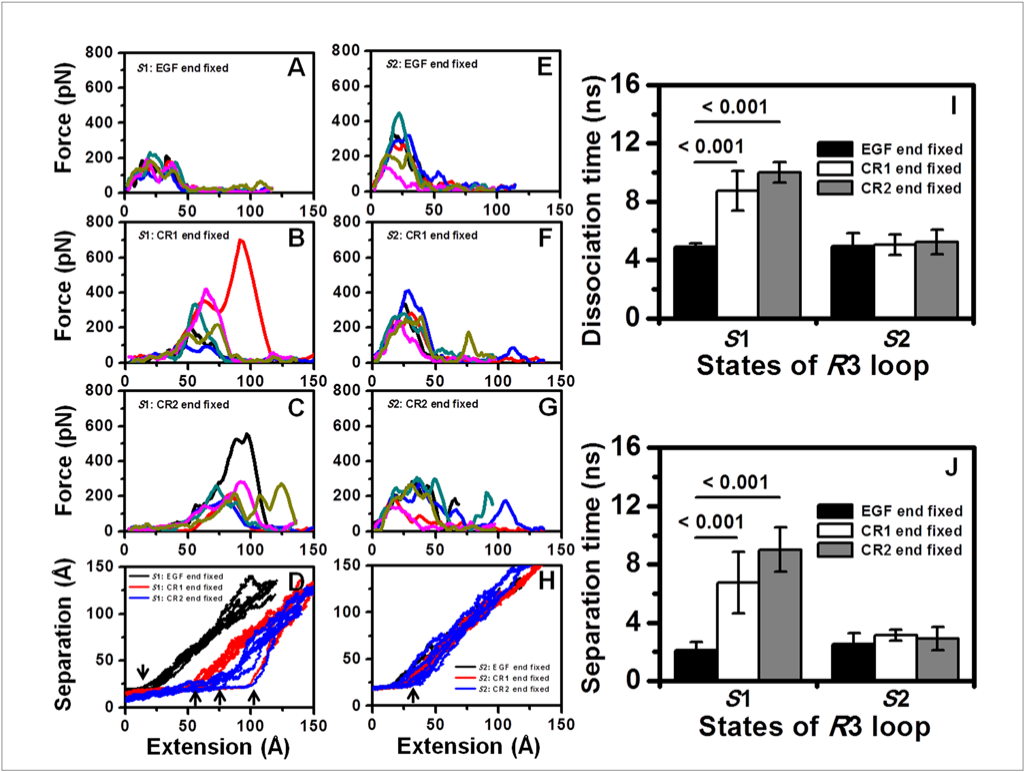
Impact of the CR domain on the constant velocity forced dissociation process. (*A*-*C*, *E*-*G*) Force-extension profiles at 0.01 Å/*ps* based on the initial conformations of the 20-*ns*-equilibrated SGP-3-ligated 2CR set of the 1G1Q system (*A*-*C*) with an *R*3 loop state of *S*1 and the 1G1S system (*E*-*G*) with an *R*3 loop state of *S*2. Three runs (*black*, *red*, *blue*) were performed for each fixation setting including EGF end fixation (*A*, *E*), CR1 end fixation (*B*, *F*) and CR2 end fixation (*C*, *G*). (*D*, *H*) The corresponding separation-extension profiles were summarized for the 1G1Q (*D*) and 1G1S (*H*) systems. Each three runs for EGF end fixation, CR1 end fixation or CR2 end fixation are shown in the same color (*black*, *red* or *blue*, respectively). (*I*, *J*) The dissociation times (*I*) and the separation time (*J*) were compared. The data are presented as the mean ± SD of six independent dissociation runs.

The force profiles of the 1G1Q/P-LECC-SGP-3 system under *cv*-0.01 Å/*ps* showed relative consistency only for the six repeats of EGF end fixation with a peak force of approximately 200 *pN* (Fig. 5*A*). Both the CR1 end-fixed (Fig. 5*B*) and CR2 end-fixed (Fig. 5*C*) simulations exhibited variable force profiles. The peak force in the second repeated process increased to ~700 *pN* (*red*) for the CR1 end-fixed simulations (Fig. 5*B*), and the first dissociation run (*black*) of the CR2 end-fixed system showed a peak force of ~ 600 *pN* (Fig. 5*C*). Accordingly, the intramolecular extension prior to essential intermolecular dissociation increased successively, from ~ 23 Å of the EGF end fixation (*black*) to ~ 50 Å for CR1 end fixation (*red*) and ~ 63 Å for CR2 end fixation (*blue*), for most dissociations of the 1G1Q/P-LECC-SGP-3 system (Fig. 5*D*, *inserted left three arrows*). The two exceptions to the force profiles (Fig. 5*B*-*C*) exhibited further intramolecular extension of ~ 95 Å (Fig. 5*D*, *inserted right arrow*). The observed intramolecular extensions were mainly due to the hinge opening of P-selectin during the normal dissociation process with intact domain structures (S8 Fig., *A*, *D*), but the EGF domain was disrupted in the two exceptions (S8 Fig., *B*, *C*). In contrast, the dissociation forces of the 1G1S/P-LECC-SGP-3 system did not show a significant difference between repeated runs of each system or between EGF end-fixed (Fig. 5*E*), CR1 end-fixed (Fig. 5*F*) and CR2 end-fixed (Fig. 5*G*) simulations with the peak force values fluctuating approximately 300 *pN*, and the corresponding separation-extension profiles displayed similar coincident transitions of approximately ~ 30 Å for all EGF (*black*), CR1 (*red*) and CR2 (*blue*) end-fixed dissociations of the 1G1S/P-LECC-SGP-3 system (Fig. 5*H*, *inserted arrow*) with intact domain structures.

The dissociation times were extended significantly for both the CR1 end-fixed (*white*) and CR2-end fixed (*gray*) simulations compared with the EGF end-fixed (*black*) simulations of the 1G1Q/P-LECC-SGP-3 system (Fig. 5*I*, *left three bars*). The results for the 1G1S/P-LECC-SGP-3 system showed comparable dissociation times among the EGF end-fixed (*black*), CR1 end-fixed (*white*) and CR2 end-fixed (*gray*) simulations (Fig. 5*I*, *right three bars*). The transition time statistics for intermolecular separation (Fig. 5*D*, *H*, *inserted arrows*) revealed the same trends as those for dissociation time (Fig. 5*J*), which indicated that the intramolecular extension behavior determined the forced dissociation of the P-selectin-PSGL-1 bond under a constant velocity of 0.01 Å/*ps*. It was understandable that the bent orientation of the EGF domain and the following CR domains of the 1G1Q/P-LECC-SGP-3 system would dissipate the external energy and protect the complex from unbinding through self-extension, resulting in a prolonged bond dissociation time. By contrast, the extended orientation of the 1G1S/P-LECC-SGP-3 system would transfer the external force effectively to the binding surface, resulting in a shortened bond lifetime.

**Fig 6:**
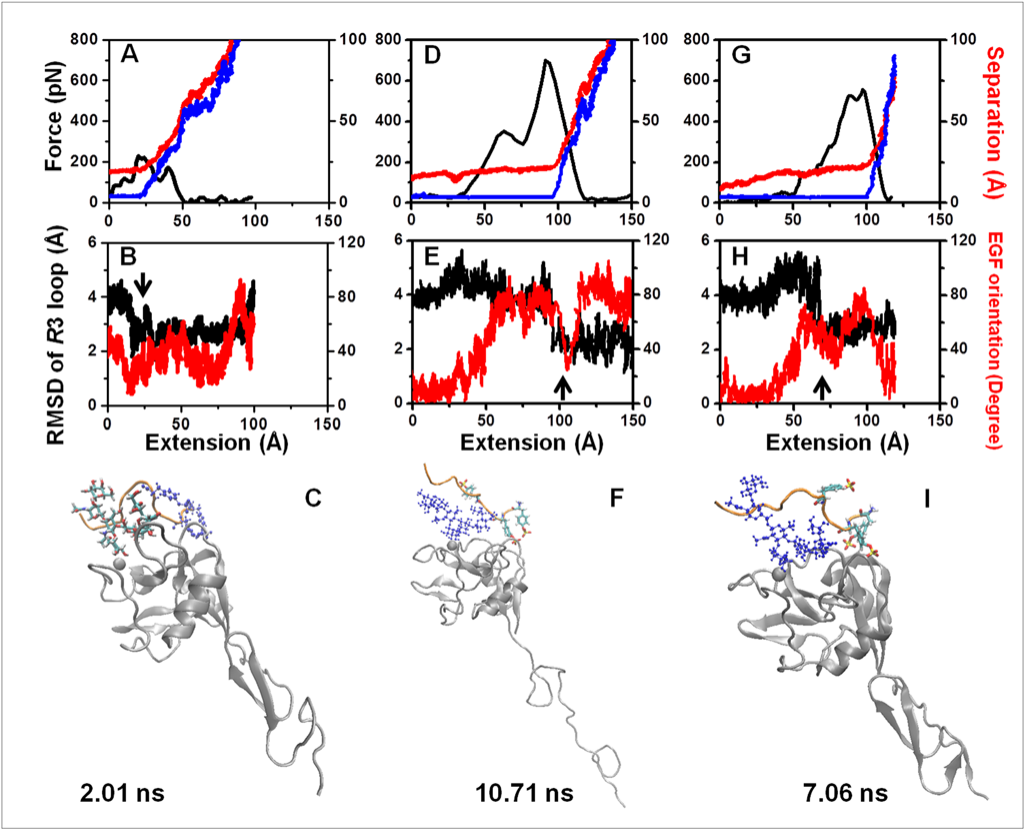
Typical features of the constant velocity forced dissociation process in the SGP-3-ligated 1G1Q 2CR complex system. The typical dissociation processes for EGF end fixation (*A-C*), CR1 end fixation (*D-F*) and CR2 end fixation (*G-I*) are shown. (*A*, *D*, *G*) The profiles of force-extension (*black*), receptor-ligand separation-extension (*red*), and Fuc-calcium ion separation-extension (*blue*) in the dissociation processes at 0.01 Å/*ps*. (*B*, *E*, *H*) Corresponding evolution of the *R*3 loop conformation (*black*) and EGF orientation (*red*). The former was quantified according to the RMSD of the *R*3 loop for the *S*1’ state based on alignment of the rigid parts of the Lec domain, and the latter was calculated based on the reference of crystallized 1G1Q. (*C*, *F*, *I*) Conformational features immediately after the critical moment of the *R*3 loop RMSD. Only P-LE (*silver*, *Newcartoon*), calcium ion (*silver*, *VDW*) and the SGP-3 ligand are presented for clarity. The peptide, Core-2 sugar, and three sulfonated tyrosines of the SGP-3 ligand are shown using the *orange newcartoon*, *blue CPK* and *name licorice* formats, respectively.

The conformational evolution of the dissociation processes clearly showed that the allosteric shift of the Lec domain *R*3 loop from the *S*1 to *S*1’ state occurred obviously twelve times in the total of eighteen dissociation processes in the 1G1Q/P-LECC-SGP-3 system, and in the six of those twelve times, allostery occurred right after complete unbinding of the SGP-3 ligand. This kind of allostery might have resulted from perturbation of the EGF domain and liberation of the Lec domain *R*3 loop from ligand binding. In the other six dissociation runs, the allostery of Lec domain *R*3 loop occurred before complex dissociation, and only one run for EGF end-fixed system, two and three runs for CR1 end-fixed and CR2 end-fixed systems, respectively. Typical allosteric dynamics for each system were exemplified in Fig. 6. Although they were similar, with the position of the force peak (Fig. 6*A*, *D*, *G*, *black*) corresponding to the transition of complex unbinding (Fig. 6*A*, *D*, *G*, *red* and *blue*) and the extension of the EGF domain (Fig. 6*B*, *E*, *H*, *red*) from the initial bent orientation occurred earlier than the allosteric shift (Fig. 6*B*, *E*, *H*, *black*) of the Lec domain, the allosteric shift took place differently. Because the external force acted on the EGF domain directly in the EGF end-fixed dissociation, the EGF orientation was disturbed immediately, which resulted in fast allostery of Lec domain R3 loop without intra-structural destroy of EGF domain (Fig. 6*A*-*C*). On the other hand, the force on EGF domain could transfer to the binding pocket quickly and resulted in short lifetime with low occurrence frequency of allostery in Lec domain before complex dissociation. Comparatively speaking, the existence of CR domains would slow the disturbance of external force on the EGF domain as well as on the binding pocket, which increased the available time for the response of the Lec domain to the change in EGF orientation and resulted in high occurrence frequency of Lec domain allostery in CR1 end-fixed (Fig. 6*D*-*F*) and CR2 end-fixed (Fig. 6*G*-*I*, S3 Movie) dissociations. The allosteric differences of occurrence frequency before complex dissociation as well as the occurrence moment in different end-fixed simulations indicated that the CR domain could also regulate the allostery of the Lec domain during forced dissociation of P-selectin-PSGL-1 interactions by modulating the EGF domain orientation. The 1G1S/P-LECC-SGP-3 system simulations did not reveal this type of difference between the EGF end-fixed, CR1 end-fixed and CR2 end-fixed dissociation processes with a relatively linear orientation of the EGF domain and the following CR domains.

### Impact of the CR domain on the cf forced dissociation processes of P-selectin-PSGL-1 complex

**Fig 7:**
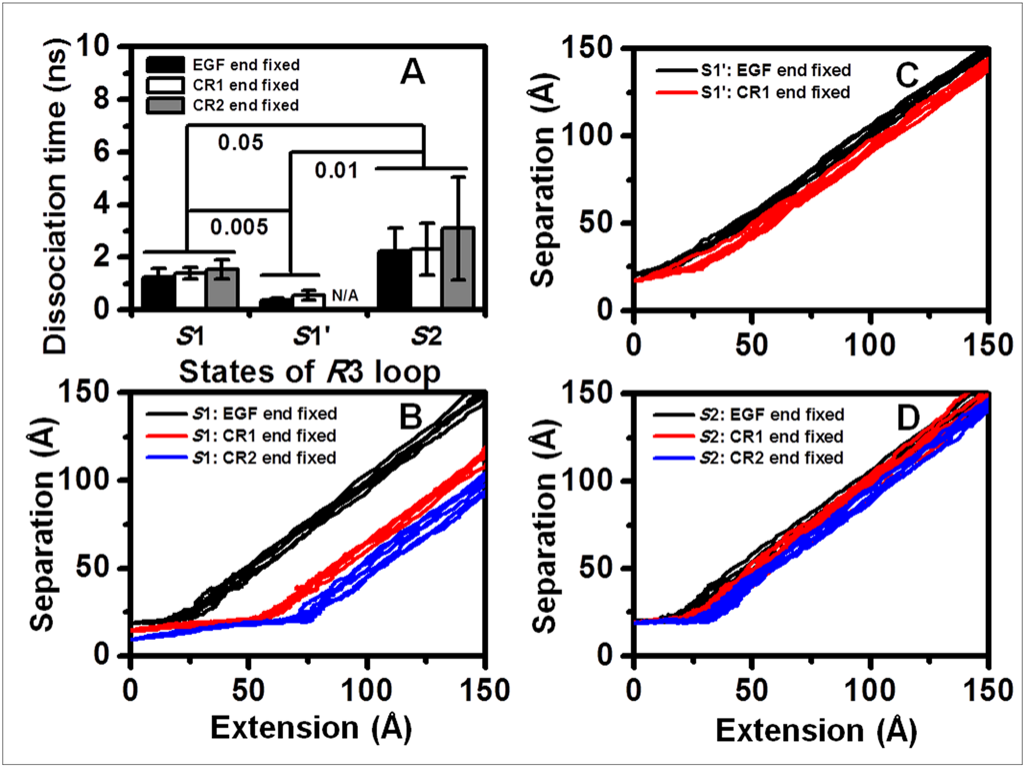
Impact of the CR domain on the constant force dissociation process. (*A*) Dissociation time distribution for the EGF end-fixed (*black*), CR1 end-fixed (*white*) and CR2 end-fixed (*gray*) dissociation processes at 300 *pN* based on 20-*ns*-equilibrated snapshots of the SGP-3-ligated 1G1Q 2CR complex with the *R*3 loop in the *S*1 state (*left*), the 1G1Q 1CR complex with the *R*3 loop in the *S*1’ state (*middle*) and the 1G1S 2CR complex with the *R*3 loop in the *S*2 state (*right*), respectively. The data are presented as the mean ± SD of six independent runs for each fixation setting. (*B*, *C*, *D*) Corresponding separation-extension profiles of the dissociation processes based on the initial *S*1 (*B*), *S*1’ (*C*) or *S*2 (*D*) system. Six repeated runs for EGF end fixation, CR1 end fixation and CR2 end fixation are shown in the same color (*black*, *red* or *blue*, respectively).

In contrast, the dissociation processes observed under a constant force of *cf*-300 *pN* did not reflect the difference between the EGF end-fixed simulations and the CR1 end-fixed or CR2 end-fixed simulations for all systems. The dissociation times were similar for the 1G1Q/P-LECC-SGP-3 system with an initial conformation of the *S*1 state (Fig. 7*A*, *left three bars*), the 1G1Q/P-LEC-SGP3 system with an initial conformation of the *S*1’ state (Fig. 7*A*, *middle two bars*), and the 1G1S/P-LECC-SGP-3 system with an initial conformation of the *S*2 state (Fig. 7*A*, *right three bars*). Combined with the finding that the peak forces under *cv*-0.01 Å/*ps* forced dissociation processes were mostly below 300 *pN* for the 1G1Q/P-LECC-SGP-3 system but fluctuated approximately 300 *pN* for the 1G1S/P-LECC-SGP-3 system (Fig. 5*A*-*B*, *E*-*G*), these data indicated that the work applied by the external force of 300 *pN* exceeded the potential barriers of the first two systems with consistent dissociation times and was comparable with that of the last system, with a high variation of dissociation times. The shorter dissociation times of the 1G1Q/P-LEC-SGP-3 system with an *S*1’ state resulted from the weaker receptor-ligand interaction (Fig. 2*C*). Thus, the difference in dissociation times observed under *cf*-300 *pN* represented the difference in the potential receptor-ligand interaction barrier for the three systems. Therefore, it was predictable that the regulatory role of the CR domain in dissociation should be investigated under the smaller force. The relationship between complex separation and intramolecular extension showed similar trends to those of unbinding processes under *cv*-0.01 Å/*ps*. The existence of the CR domain would increase the intramolecular extension more apparently for the 1G1Q/P-LECC-SGP-3 system (Fig. 7*B*) than for the 1G1S/P-LECC-SGP-3 system (Fig. 7*D*). The rapid dissociations due to weak receptor-ligand interactions resulted in decreased intramolecular extensions prior to essential dissociation for the 1G1Q/P-LEC-SGP-3 system with an initial Lec domain conformation of the *S*1’ state (Fig. 7*C*).

Combined with the *cv*-0.01 Å/*ps* and *cf*-300 *pN* simulations, the results suggested that the CR domain could mediate the forced dissociation of P-selectin-PSGL-1 interactions in two ways. First, the CR domain could slow the dissociation process through intramolecular extension. Second, the existence of the CR domain could increase the possibility of Lec domain allostery during the dissociation process. However, the requirement is that the external force should be appropriately low for a sufficient response of the complex to occur.

## Discussion

Relative to the Lec and EGF domains, the contribution of the CR domain to the conformational dynamics of selectins is unclear. By combining MD simulations and homology modeling, we investigated the impact of the CR domain on the micro-structural features of P-selectin in this study. The results showed that the CR domain tended to stabilize the low affinity of P-selectin during the equilibration processes with the transition inhibition from the *S*1 to *S*1’ state by restraining the extension of the bent EGF orientation, or with the relaxation acceleration of the *S*2 state by promoting the bending of the extended EGF orientation (Figs. 3–4, S4–S7 Figs.). Furthermore, the existence of the CR domain increased intramolecular extension prior to essential unbinding, resulting in an extended bond dissociation time during the forced unbinding process. The behavior would increase the allosteric potential of the Lec domain to shift from *S*1 to *S*1’ during the dissociation process by increasing the available time for Lec domain to respond to the change of EGF domain orientation (Figs. 5–7). These results further confirmed the regulatory role of the EGF domain in determining the conformation of the Lec domain as well as the P-selectin-ligand interactions.

The allostery of the P-selectin Lec domain and the regulation of allostery through the orientation of the EGF domain have been proposed upon experiments [18] and also predicted from crystallized structure analyses [19]. The allostery model for selectins is a widely accepted model put forth to explain the micro-structural mechanism of the counterintuitive catch bond behavior observed in all selectin members [29], that is, selectin-ligand bonds dissociate less rapidly at higher forces than at lower forces, which plays key roles in the biological function of flow-enhanced cell adhesion [30–32]. The spontaneous transition of the EGF domain between bent and extended orientations and the close relationship between the EGF domain orientation and Lec domain conformation found in this study intuitively confirmed the allostery of P-selectin at the atomic level. The ability of the EGF domain to regulate the conformation of the Lec domain depends on complicated hinge interaction between the EGF domain and the Lec domain as well as the ability to adopt different orientations spontaneously, which would reset the Lec-EGF hinge interaction and trigger the allosteric change in the Lec domain through long distance correlation between the Lectin and EGF domains. The first CR domain could regulate the allosteric shift of the Lec domain by adjusting the tendency of the EGF orientation. This function is conferred by the unique feature of the EGF-CR1 hinge interaction that is weaker than that of Lec-EGF hinge but stronger than that of the CR1-CR2 hinge (S9 Fig.). The moderate hinge interaction would enable the first CR domain to regulate the orientation of EGF through its own movement. Additional CR domains would constrain the freedom of the CR1 domain and decrease its impact on the EGF domain. This unique feature of the first CR domain has a corresponding structural basis. Sequence alignment of nine P-selectin CR domains revealed that the first CR domain lacked the conserved Ala amino acid residue, which was replaced by Val (S2 Table, *A*, *left red column*). This means that the first CR domain showed less independence than the other CR domains because Ala is the second smallest amino acid with a short side chain. The first CR domain uniquely exhibits two charged amino acids (Arg and Glu) immediately after the Val residue, which would increase the hinge interaction between the first CR domain and the EGF domain. The reduction of independence and the increase in the hinge interaction would enhance the ability of the first CR domain to regulate the EGF domain orientation and, moreover, the Lec domain conformation through its own movement. The observed regulation of allostery by the first CR domain was consistent with the experimental observation whereby increasing the flexibility of the Lec-EGF domain hinge induces an earlier transition from a catch bond to a slip bond, as the changed flexibility of the EGF domain induced by the CR1 domain was equivalent to the changed hinge flexibility of the EGF-Lec domain induced by hinge mutant [17]. In fact, CR domains are functioned in providing ligand binding specificity, in addition to physical support, which was observed many years ago in experiments showing that L-selectin with an E-selectin CR domain binds HL-60 cells much better than L-selectin with a P-selectin CR domain, despite the presence of fewer CR domains [33].

The occurrence of intramolecular extension prior to intermolecular dissociation during forced dissociation processes was predicted through MD simulations using P-LE systems for both hinge opening between the Lec and EGF domains and disruption of the secondary structure of the EGF domain [25,34]. While the contribution of this intramolecular extension to intermolecular dissociation is considered to increase the probability of sliding-rebinding of the ligand and, furthermore, to extend the bond lifetime [34], the simulations performed in the current study indicated that the allostery in the Lec domain could take place during the dissociation process if the complex had sufficient time to respond to the intramolecular change. The existence of CR domains favors the allosteric change during the forced dissociation process when there is increased time available for intramolecular extension. Furthermore, the occurrence of allostery during the dissociation process would prolong the bond lifetime. But whether, when and how the allostery takes place depends on the force history [35]. A large abrupt, constant force would overcome the energy barrier easily, resulting in rapid dissociation, without the opportunity for allostery. Gradually increased forces would help the complex to adjust the regulated energy barrier and undergo the conformational change. Therefore, it is understandable that different dissociation loading rates can overcome different energy barriers by changing the responding coordinates under the dynamic force spectroscopy (DFS) theory [36], and the “jump/ramp” mode would show different dissociation pathways [37].

Conclusively, this study further confirmed the allostery of P-selectin and the existence of a transition state, *S*1’, between *S*1 and *S*2. Our findings also revealed the regulatory ability of the EGF domain in relation to allostery through changes in its orientation and its long distance correlation with the Lectin domain, and predicted the function of the CR domain in the P-selectin allosteric shift during both equilibration and dissociation processes. Our results further the understanding of the structure-function relationship of P-selectin in the viewpoint of CR presence.

## Supporting Information

S1 Fig Orientation definitions of EGF and CR1 domains. (*A*) The EGF orientation was defined by the relative angle of the target EGF domain to the reference EGF domain based on the alignment of the rigid parts of the Lec domain, which was quantified using the two vectors connecting the geometric center of the heavy atoms of the Lec-EGF hinge (residues A120 and S121) of reference structure to that of main EGF domain (residues C122 to T141) of reference structure (*blue*) and the target structure (*silver*). (*B*) The CR1 orientation was defined by the relative angle of the target CR1 domain to the reference CR1 domain based on the alignment of the EGF domain, which was indicated by the angle between the two vectors connecting the geometric center of the heavy atoms of the EGF-CR1 hinge (residues C159 and G160) of the reference CR1 domain to that of the CR1 domain (residues E161 to L217) of the reference (*blue*) and the target (*silver*) structures.
(TIF)

S2 Fig Structural features and reliability assessment of the homology-modeled human P-selectin CR1 domain and EGF-CR1 linkage. (*A*) Conformational consistency among the homology-modeled structures of the first CR domain of human P-selectin upon different templates, which was quantified using the backbone C_α_ distances of every residue between the reference based on the template of 2RLQ and each of other five structures. (*B*) A typical conformation of the P-selectin first CR domain modeled based on the template of 2RLQ. The first residue of the N terminus and the last residue of the C terminus were presented in *CPK* format with a distance of 34.6 Å between their C_α_ atoms, and the β-sheet, loop and turn are illustrated in *yellow*, *cyan* and *blue*, respectively. (*C*) Conformational comparison between modeled P-selectin CR1 domain upon 2RLQ template (*cyan*) and crystallized E-selectin CR1 domain (*purple*), three disulfide bonds were highlighted in *CPK* and *licorice*, respectively. (*D*) Conformational difference between crystallized E-selectin CR1 (*purple*) and CR2 (*silver*) domains. (*E*) Orientation consistency of CR1 domain relative to EGF domain between modeled P-selectin (*cyan*) and crystallized E-selectin (*purple*) upon alignment of EGF domains. All structures were presented as in the *NewRibbons* format. The conformational comparisons (*A*, *C*-*E*) were all upon the backbone atom alignments of target regions. The E-selectin structure was adopted from PDB code of 4CSY.
(TIF)

S3 Fig Characteristics of the conformational dynamics of the P-selectin Lec and EGF domains. (*A*) Crystallized unligated (*silver*) (PDB code: 1G1Q) and SGP-3-ligated (*blue*) (PDB code: 1G1S) P-LE domains are superposed and are presented in *newcartoon* format. The loop from P81 to D89 is labeled as the *R*3 loop, and the calcium ion is illustrated in *VDW*. (*B*) The conformational differences in the *R*3 loop between 1G1Q and 1G1S are highlighted in *topview* for the Lec domain and are labeled as *S*1 and *S*2, respectively. (*C*, *D*) The conformational dynamics of the *R*3 loop during free equilibrations of P-LE domains from 1G1Q (*C*) and 1G1S (*D*) are quantified based on RMSD evolution *via* alignment to both 1G1Q (*black*) and 1G1S (*blue*). The conformational features are illustrated by superposing the typical 30 *ns* snapshots (*E*, *F*) or final states (*G*) with the original conformations of 1G1Q (*silver*) and 1G1S (*blue*). The conformational superposition shown in (*A*-*B*, *E*-*G*) and the RMSD calculations for (*C*-*D*) are all based on alignment of the alpha carbon atoms of the rigid regions of the Lec domain. The Lec domain, except for the *R*3 loop and calcium ion, is presented in *transparent* format for clarity in (*B*, *E*-*G*).
(TIF)

S4 Fig Conformational dynamics of the unligated 1G1Q systems of the 0CR set during equilibration. The RMSD of the Lec domain *R*3 loop with respect to the reference *S*1 (*black*), *S*1’ (*red*) and *S*2 (*blue*) states (*left column*), and the EGF orientation with respect to the references of crystallized 1G1Q (*black*) and 1G1S (*red*) (*right column*), were quantified for each of six repeated runs of the 1G1Q/P-LE systems.
(TIF)

S5 Fig Conformational dynamics of the unligated 1G1Q systems of the 1CR set during equilibration. The RMSD of the Lec domain *R*3 loop with respect to the reference *S*1 (*black*), *S*1’ (*red*) and *S*2 (*blue*) states (*left column*), the EGF orientation with respect to the references of crystallized 1G1Q (*black*) and 1G1S (*red*) (*middle column*), and the CR1 orientation in relation to the reference of the respective initial conformation (*right column*) were quantified for each of six repeated runs of the 1G1Q/P-LEC systems.
(TIF)

S6 Fig Conformational dynamics of the unligated 1G1S systems of the 0CR set during equilibration. The RMSD of the Lec domain *R*3 loop with respect to the references of *S*1 (*black*), *S*1’ (*red*) and *S*2 (*blue*) states (*left column*), and the EGF orientation to the references of crystallized 1G1Q (*black*) and 1G1S (*red*) (*right column*) were quantified for each of six repeated runs of 1G1S/P-LE systems.
(TIF)

S7 Fig Conformational dynamics of the unligated 1G1S systems of the 1CR set during equilibration. The RMSD of the Lec domain *R*3 loop with respect to the references of *S*1 (*black*), *S*1’ (*red*) and *S*2 (*blue*) states (*left column*), the EGF orientation to the references of crystallized 1G1Q (*black*) and 1G1S (*red*) (*middle column*), and the CR1 orientation to the reference of respective initial conformation (*right column*) were quantified for each of six repeated runs 1G1S/P-LEC systems.
(TIF)

S8 Fig Intramolecular extension and intermolecular separation of the 1G1Q/P-LECC-SGP-3 system under *cv* 0.01 Å/ps forced dissociation. (*A*, *B*) First (*A*) and second (*B*) runs of the CR1 end-fixed dissociation process. (*C*, *D*) First (*C*) and second (*D*) runs of the CR2 end-fixed dissociation process.
(TIF)

S9 Fig Comparison of the nonbond interaction energy between the Lec-EGF hinge, EGF-CR1 hinge and CR1-CR2 hinge. The interaction energy of each trajectory was averaged first, and the results were then averaged over the different equilibration simulation runs. Consequently the interaction energy between the Lec and EGF domains was the average of the first two repeated runs for all 0CR, 1CR and 2CR sets. The interaction energy of the EGF-CR1 hinge was the average of the first two repeated runs of all 1CR and 2CR sets, and that of the CR1_CR2 hinge was the average of all 2CR set. The data were presented as the mean ± SD.
(TIF)

S1 Movie The first 20-*ns* equilibration process of the 1G1Q/P-LEC system (AVI)

S2 Movie The first 20-*ns* equilibration process of the 1G1S/P-LEC system (AVI)

S3 Movie The first *cv*-0.01 Å/ps forced dissociation process of the 1G1Q/P-LECC-SGP-3 system under CR2 end-fixed. Whole molecular extension (*left*), SGP-3 ligand unbinding and allostery of the Lec domain (*middle*), and evolution of the EGF domain orientation (*right*) were highlighted, respectively.
(AVI)

S1 Table Structural evaluations of homology-modeled human P-selectin first CR domain based on different templates. Blast score represents the sequence similarity between the first CR domain of P-selectin first CR and respective template, and the other data represent the percentage of non-optimal stereochemistry structures. *: ≥ 5.0%; ^¶^: ≥10%.
(DOCX)

S2 Table (*A*) Sequence alignment of nine CR domains from human P-selectin. Fully conserved sites throughout the nine domains are indicated with *blue* boxes, and partially conserved sites are indicated with *red* boxes. Three disulfide bonds are formed in each CR domain between 1–5 cysteines, 2–3 and 4–6 cysteines. (*B*) Disulfide bond distributions for various homology modeling templates. The templates with two disulfide bond pairs which close to the human P-selectin CR domain are highlighted with *gray* boxes.
(DOCX)
