# Supplementary figures and images for: Contribution of the CR Domain to P-Selectin Lectin Domain Allostery by Regulating the Orientation of the EGF Domain
Source: PLoS One. 2015 Feb 12;10(2):e0118083. doi: 10.1371/journal.pone.0118083 (PMC4326174; doi:10.1371/journal.pone.0118083)

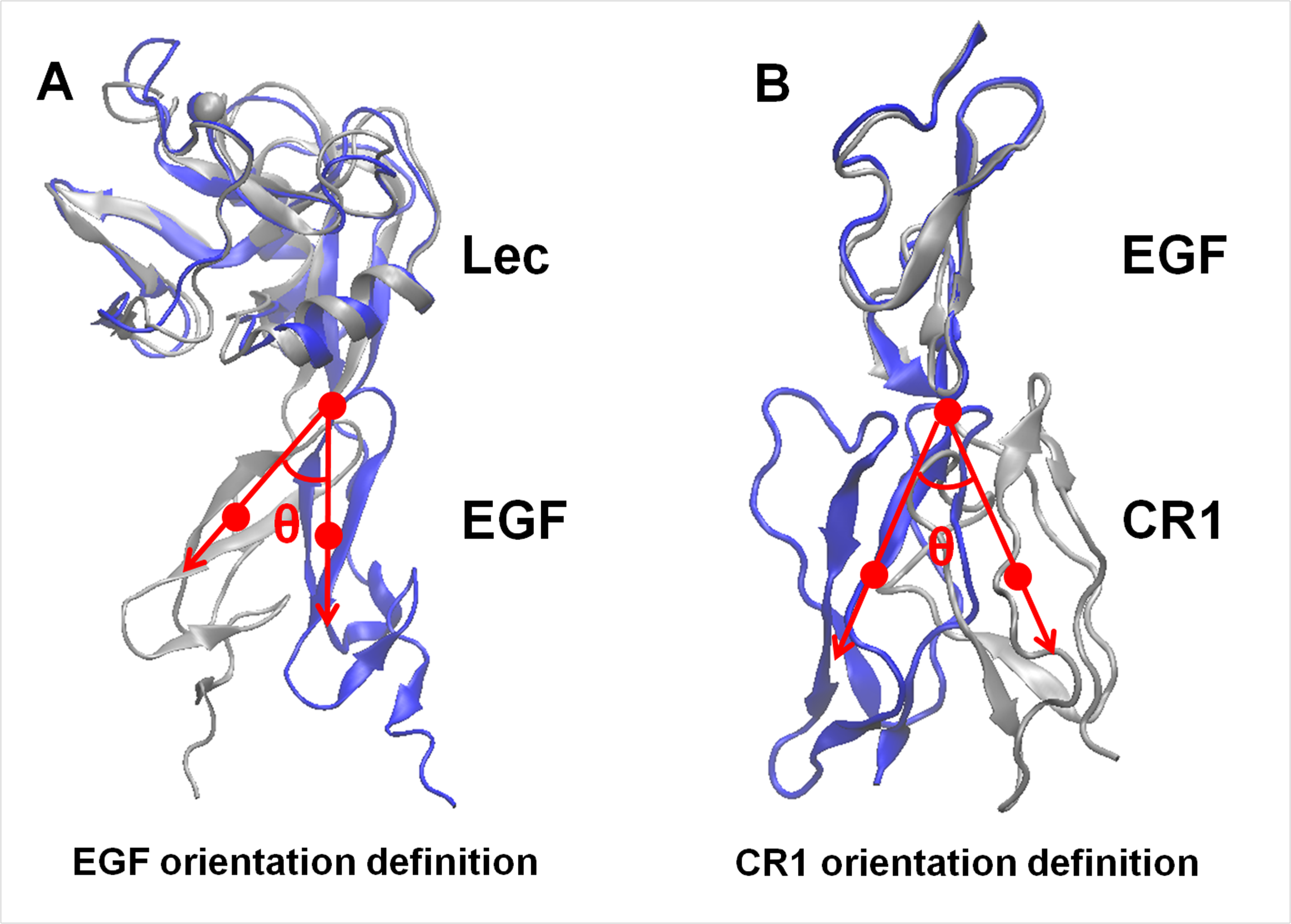

Supplement: S1 Fig — (A) The EGF orientation was defined by the relative angle of the target EGF domain to the reference EGF domain based on the alignment of the rigid parts of the Lec domain, which was quantified using the two vectors connecting the geometric center of the heavy atoms of the Lec-EGF hinge (residues A120 and S121) of reference structure to that of main EGF domain (residues C122 to T141) of reference structure (blue) and the target structure (silver). (B) The CR1 orientation was defined by the relative angle of the target CR1 domain to the reference CR1 domain based on the alignment of the EGF domain, which was indicated by the angle between the two vectors connecting the geometric center of the heavy atoms of the EGF-CR1 hinge (residues C159 and G160) of the reference CR1 domain to that of the CR1 domain (residues E161 to L217) of the reference (blue) and the target (silver) structures. (TIF) [file pone.0118083.s001.tif]

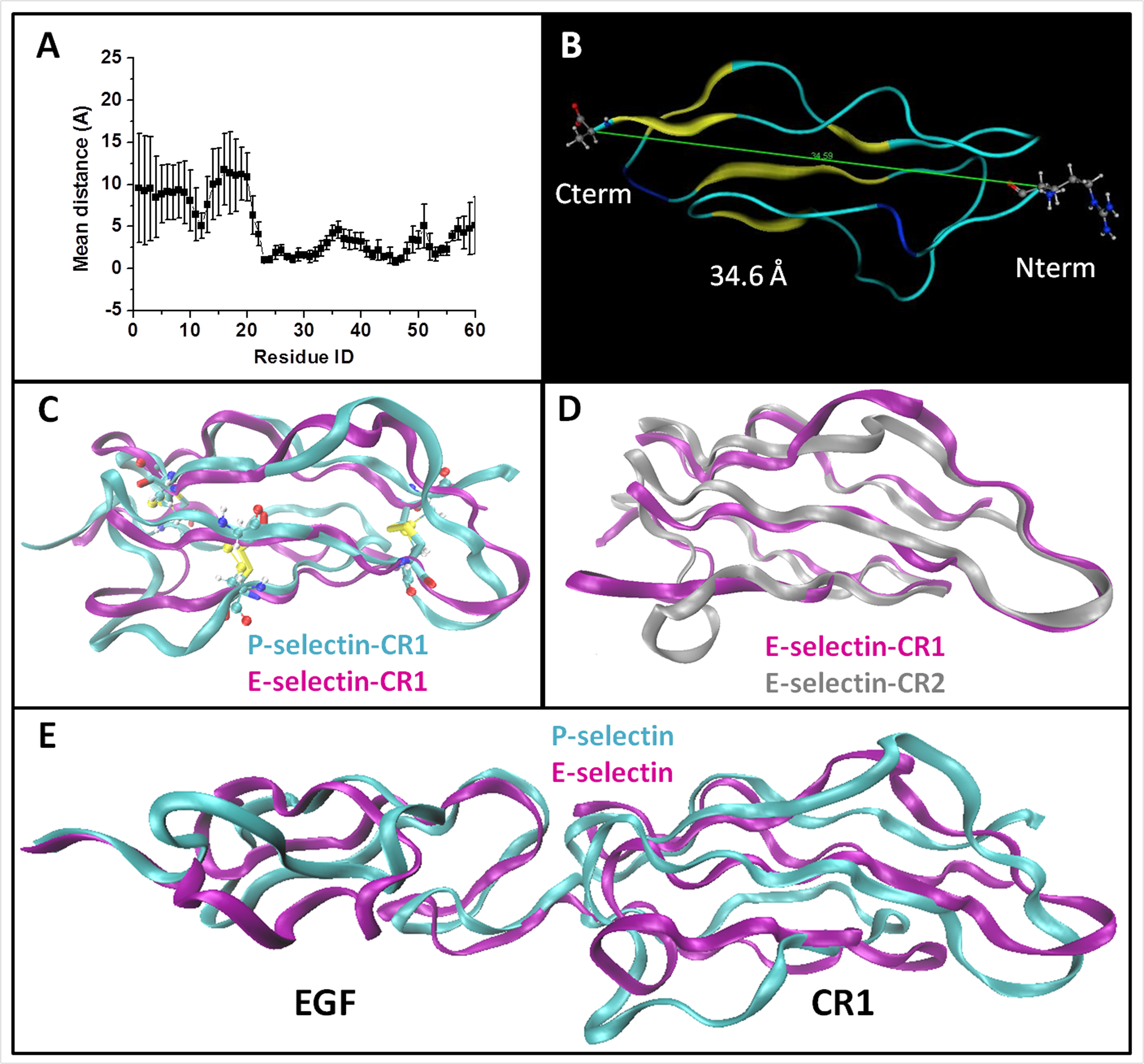

Supplement: S2 Fig — (A) Conformational consistency among the homology-modeled structures of the first CR domain of human P-selectin upon different templates, which was quantified using the backbone Cα distances of every residue between the reference based on the template of 2RLQ and each of other five structures. (B) A typical conformation of the P-selectin first CR domain modeled based on the template of 2RLQ. The first residue of the N terminus and the last residue of the C terminus were presented in CPK format with a distance of 34.6 Å between their Cα atoms, and the β-sheet, loop and turn are illustrated in yellow, cyan and blue, respectively. (C) Conformational comparison between modeled P-selectin CR1 domain upon 2RLQ template (cyan) and crystallized E-selectin CR1 domain (purple), three disulfide bonds were highlighted in CPK and licorice, respectively. (D) Conformational difference between crystallized E-selectin CR1 (purple) and CR2 (silver) domains. (E) Orientation consistency of CR1 domain relative to EGF domain between modeled P-selectin (cyan) and crystallized E-selectin (purple) upon alignment of EGF domains. All structures were presented as in the NewRibbons format. The conformational comparisons (A, C-E) were all upon the backbone atom alignments of target regions. The E-selectin structure was adopted from PDB code of 4CSY. (TIF) [file pone.0118083.s002.tif]

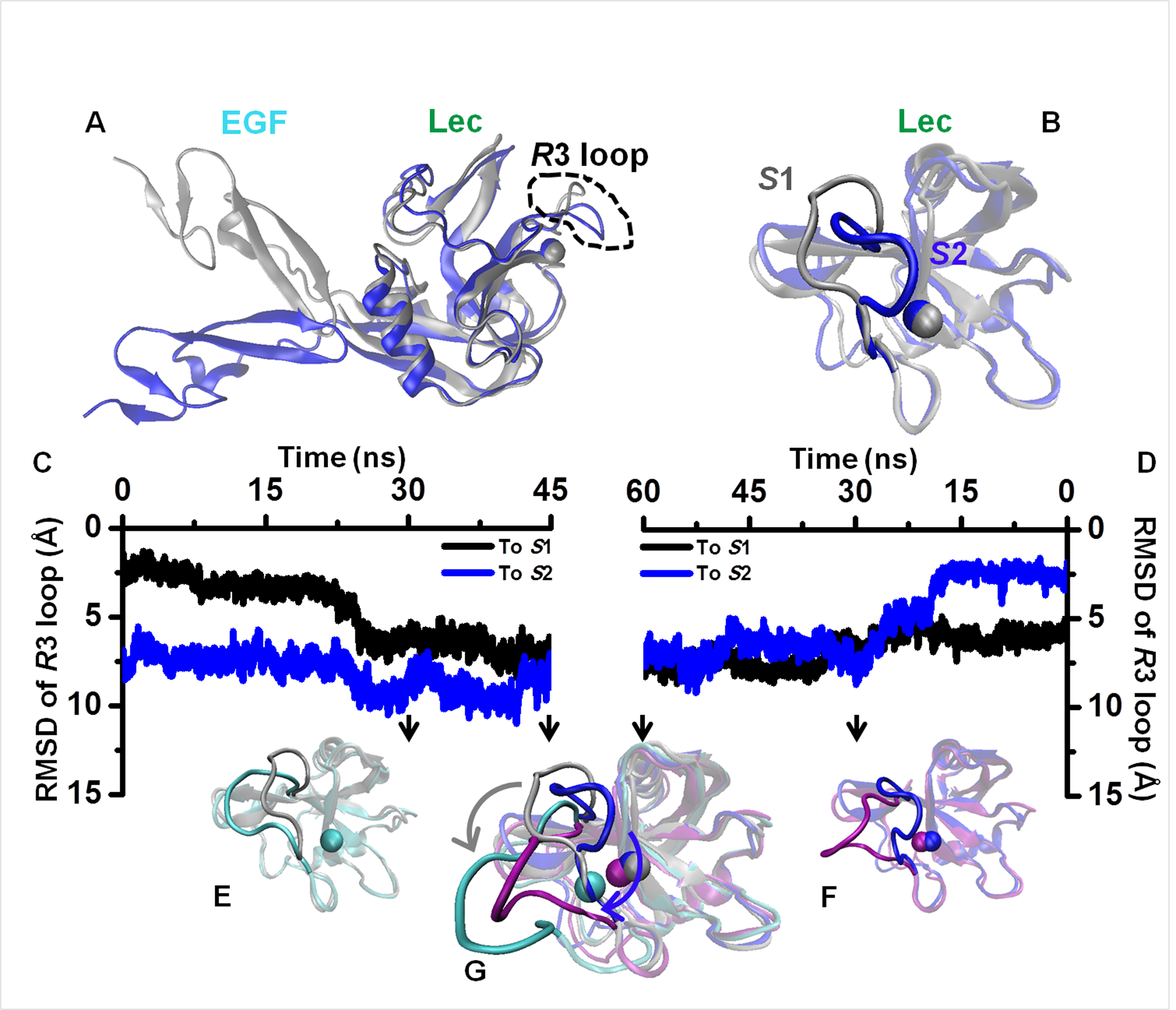

Supplement: S3 Fig — (A) Crystallized unligated (silver) (PDB code: 1G1Q) and SGP-3-ligated (blue) (PDB code: 1G1S) P-LE domains are superposed and are presented in newcartoon format. The loop from P81 to D89 is labeled as the R3 loop, and the calcium ion is illustrated in VDW. (B) The conformational differences in the R3 loop between 1G1Q and 1G1S are highlighted in topview for the Lec domain and are labeled as S1 and S2, respectively. (C, D) The conformational dynamics of the R3 loop during free equilibrations of P-LE domains from 1G1Q (C) and 1G1S (D) are quantified based on RMSD evolution via alignment to both 1G1Q (black) and 1G1S (blue). The conformational features are illustrated by superposing the typical 30 ns snapshots (E, F) or final states (G) with the original conformations of 1G1Q (silver) and 1G1S (blue). The conformational superposition shown in (A-B, E-G) and the RMSD calculations for (C-D) are all based on alignment of the alpha carbon atoms of the rigid regions of the Lec domain. The Lec domain, except for the R3 loop and calcium ion, is presented in transparent format for clarity in (B, E-G). (TIF) [file pone.0118083.s003.tif]

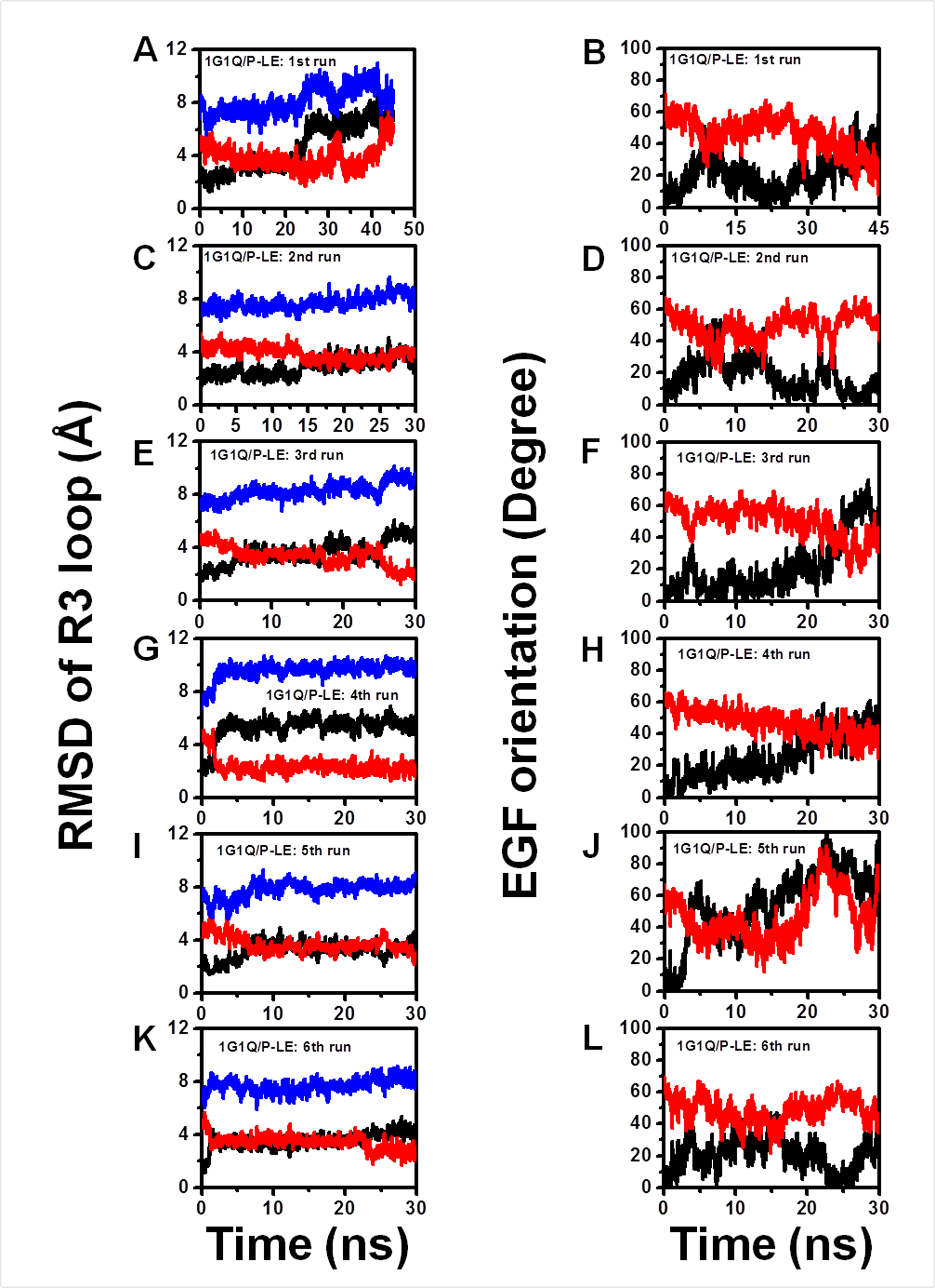

Supplement: S4 Fig — The RMSD of the Lec domain R3 loop with respect to the reference S1 (black), S1’ (red) and S2 (blue) states (left column), and the EGF orientation with respect to the references of crystallized 1G1Q (black) and 1G1S (red) (right column), were quantified for each of six repeated runs of the 1G1Q/P-LE systems. (TIF) [file pone.0118083.s004.tif]

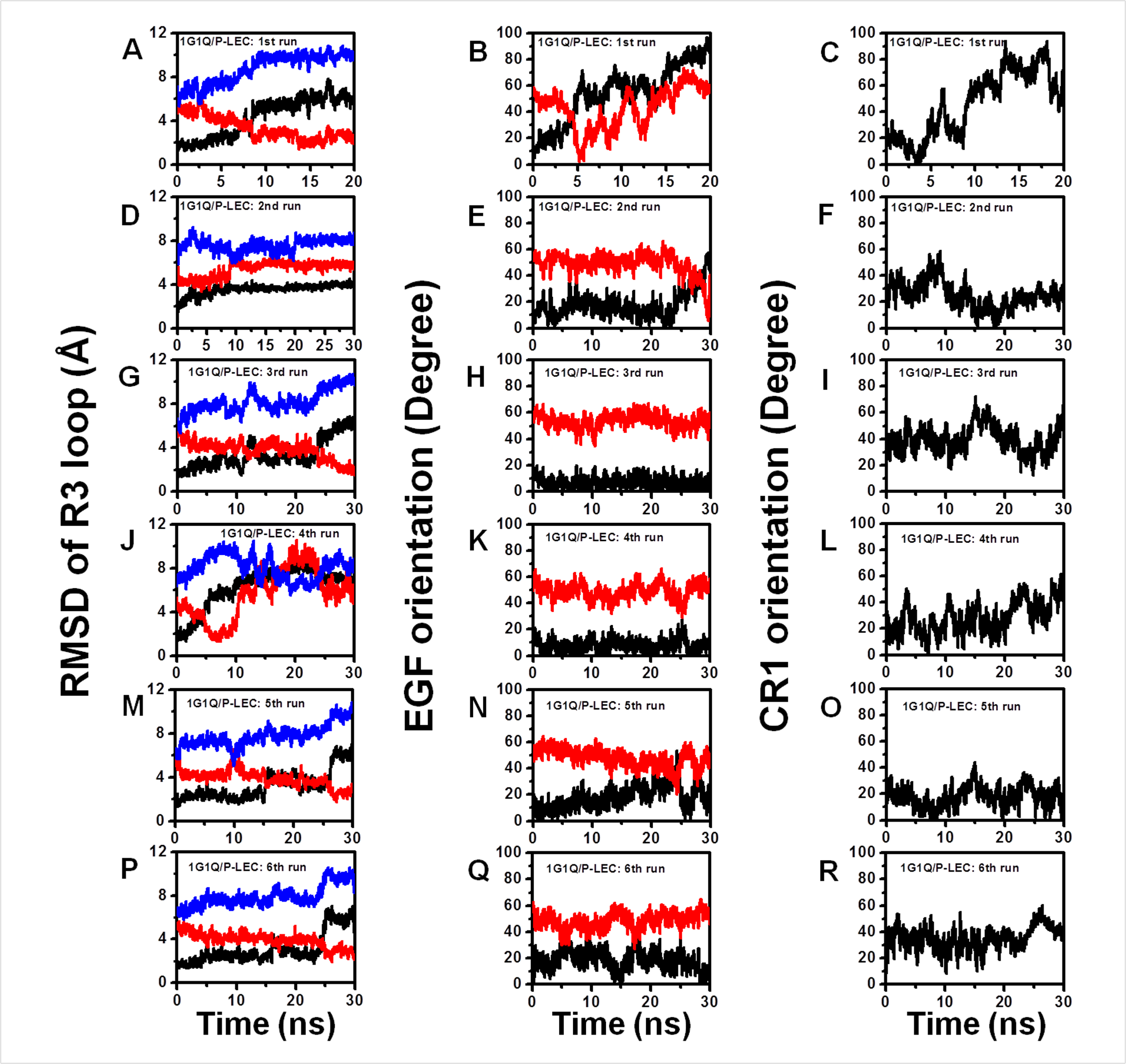

Supplement: S5 Fig — The RMSD of the Lec domain R3 loop with respect to the reference S1 (black), S1’ (red) and S2 (blue) states (left column), the EGF orientation with respect to the references of crystallized 1G1Q (black) and 1G1S (red) (middle column), and the CR1 orientation in relation to the reference of the respective initial conformation (right column) were quantified for each of six repeated runs of the 1G1Q/P-LEC systems. (TIF) [file pone.0118083.s005.tif]

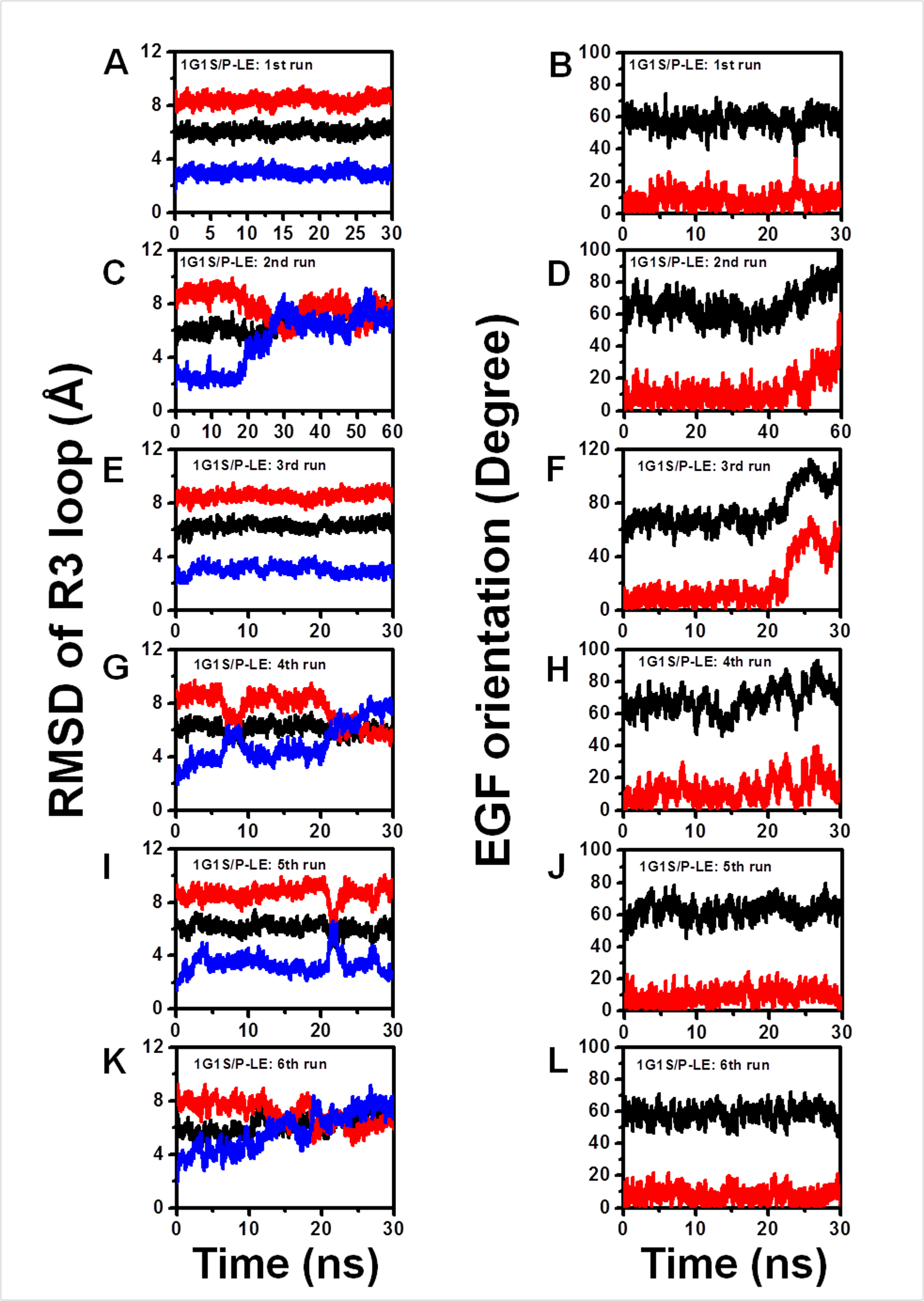

Supplement: S6 Fig — The RMSD of the Lec domain R3 loop with respect to the references of S1 (black), S1’ (red) and S2 (blue) states (left column), and the EGF orientation to the references of crystallized 1G1Q (black) and 1G1S (red) (right column) were quantified for each of six repeated runs of 1G1S/P-LE systems. (TIF) [file pone.0118083.s006.tif]

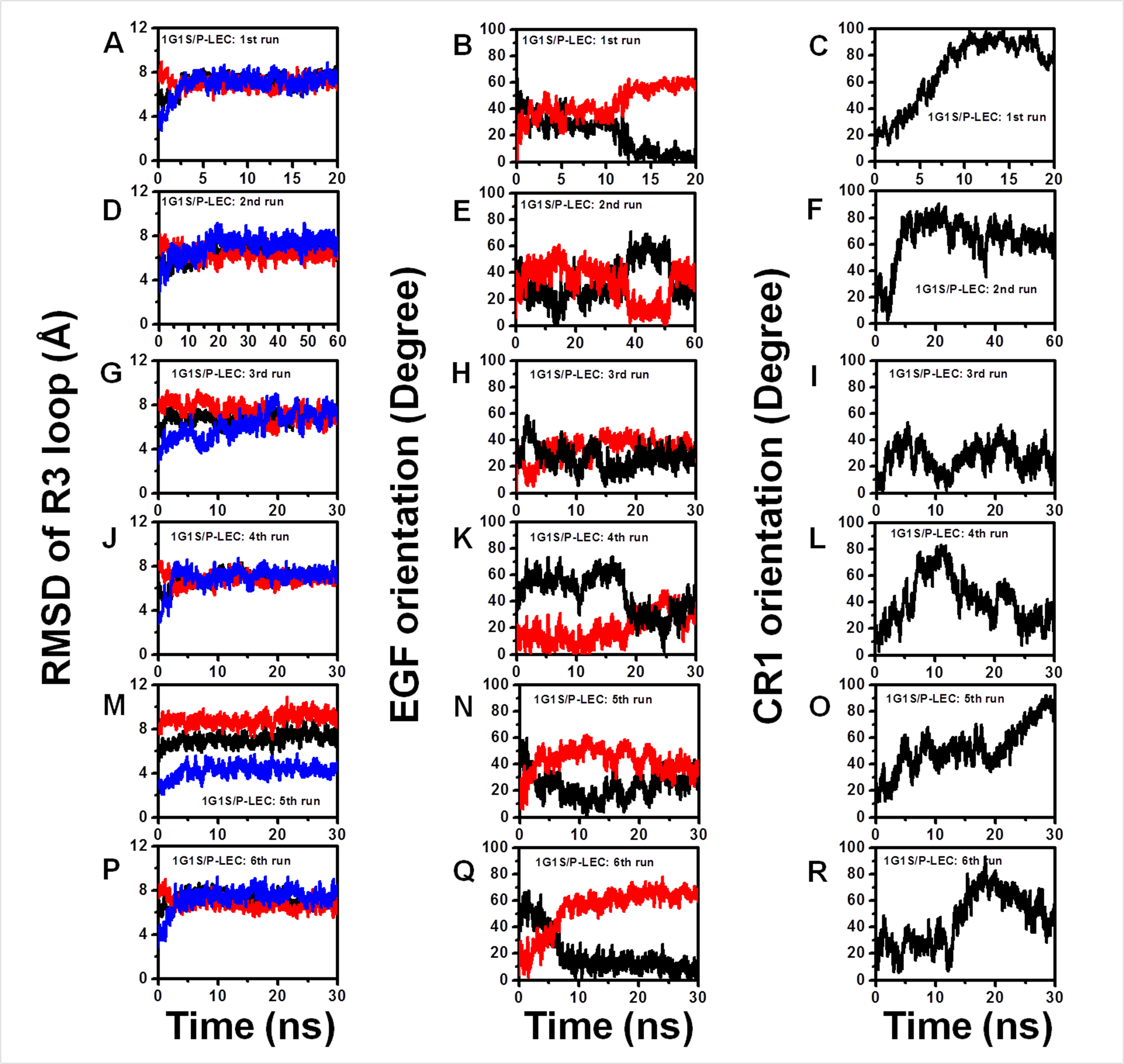

Supplement: S7 Fig — The RMSD of the Lec domain R3 loop with respect to the references of S1 (black), S1’ (red) and S2 (blue) states (left column), the EGF orientation to the references of crystallized 1G1Q (black) and 1G1S (red) (middle column), and the CR1 orientation to the reference of respective initial conformation (right column) were quantified for each of six repeated runs 1G1S/P-LEC systems. (TIF) [file pone.0118083.s007.tif]

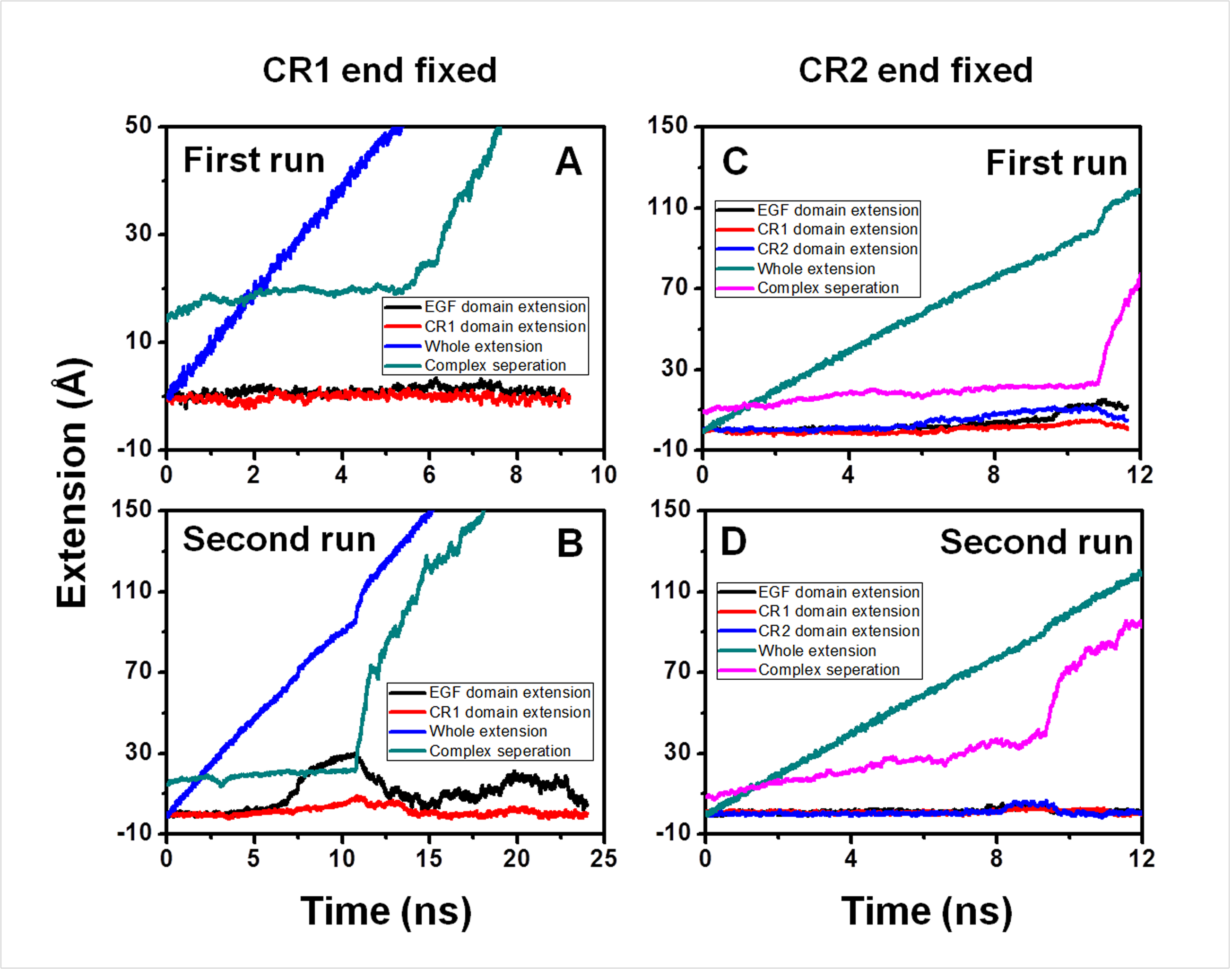

Supplement: S8 Fig — (A, B) First (A) and second (B) runs of the CR1 end-fixed dissociation process. (C, D) First (C) and second (D) runs of the CR2 end-fixed dissociation process. (TIF) [file pone.0118083.s008.tif]

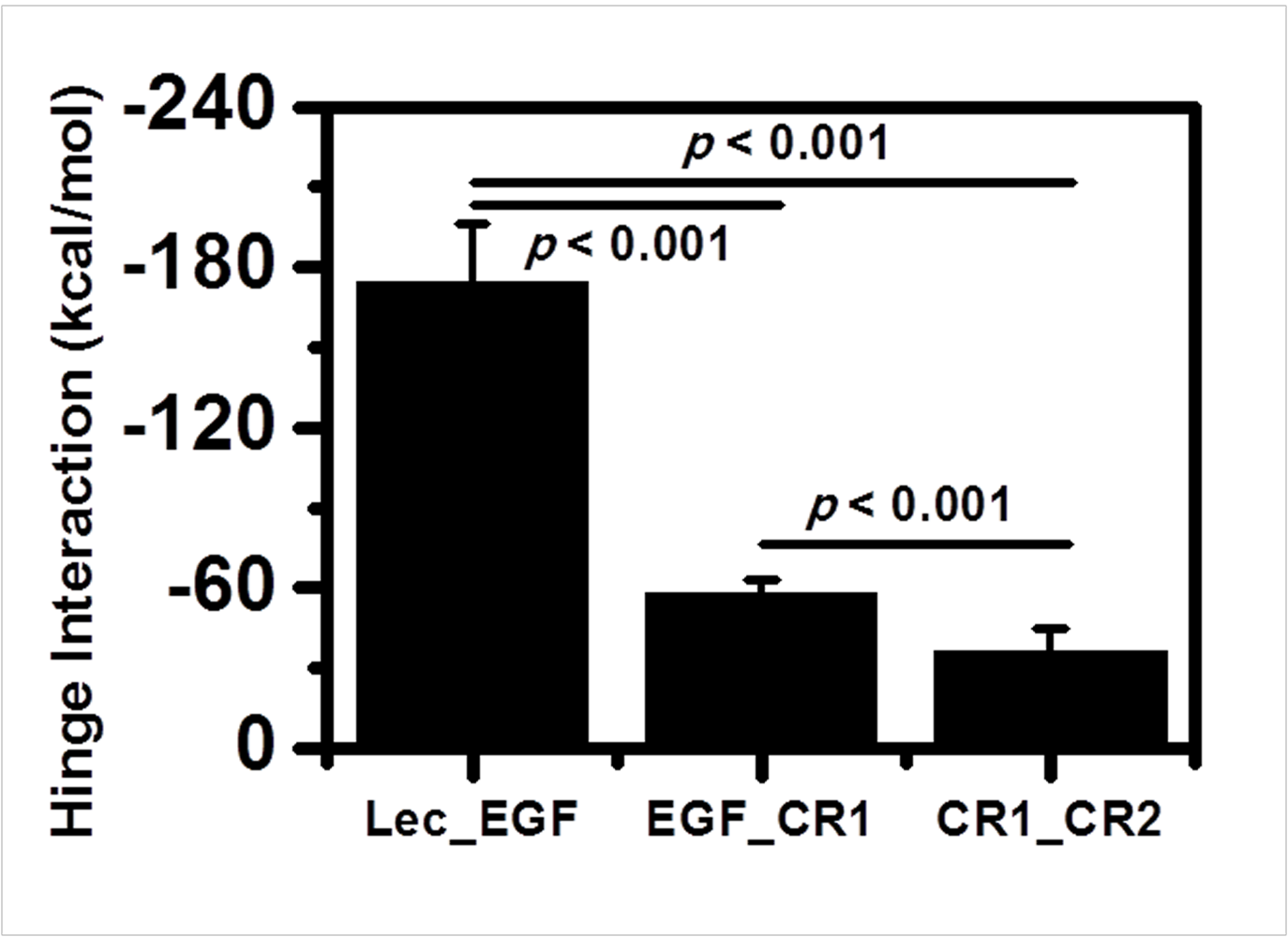

Supplement: S9 Fig — The interaction energy of each trajectory was averaged first, and the results were then averaged over the different equilibration simulation runs. Consequently the interaction energy between the Lec and EGF domains was the average of the first two repeated runs for all 0CR, 1CR and 2CR sets. The interaction energy of the EGF-CR1 hinge was the average of the first two repeated runs of all 1CR and 2CR sets, and that of the CR1_CR2 hinge was the average of all 2CR set. The data were presented as the mean ± SD. (TIF) [file pone.0118083.s009.tif]
