# Supplementary material for: Contribution of the CR Domain to P-Selectin Lectin Domain Allostery by Regulating the Orientation of the EGF Domain
Source: PLoS One. 2015 Feb 12;10(2):e0118083. doi: 10.1371/journal.pone.0118083 (PMC4326174; doi:10.1371/journal.pone.0118083)
Supplement: S1 Table — Blast score represents the sequence similarity between the first CR domain of P-selectin first CR and respective template, and the other data represent the percentage of non-optimal stereochemistry structures. *: ≥ 5.0%; ¶: ≥10%. (DOCX) [file pone.0118083.s013.docx]

| Templates | Blast score | (Φ, Ψ)^†^ | Bond lengths | Bond angles | Dihedrals | Rotamers | Atom clashes |
| --- | --- | --- | --- | --- | --- | --- | --- |
| 2G7I | 27.7 | 3.3 | 4.3 | 2.6 | 1.3 | 3.7 | 0 |
| 1RID | 26.9 | 5.0^*^ | 5.9^*^ | 6.3^*^ | 2.7 | 0 | 0 |
| 1OK3 | 28.5 | 3.3 | 4.3 | 3.3 | 1.3 | 0 | 0 |
| 1GKN | 34.7 | 5.0^*^ | 10.2^¶^ | 7.6^*^ | 2.3 | 3.7 | 0 |
| 2RLQ | 36.2 | 3.3 | 5.3 * | 4.0 | 2.0 | 0 | 0 |
| 1PPQ | 35.4 | 3.3 | 8.6^*^ | 6.3^*^ | 1.7 | 0 | 0 |
