# Supplementary material for: Contribution of the CR Domain to P-Selectin Lectin Domain Allostery by Regulating the Orientation of the EGF Domain
Source: PLoS One. 2015 Feb 12;10(2):e0118083. doi: 10.1371/journal.pone.0118083 (PMC4326174; doi:10.1371/journal.pone.0118083)
Supplement: S2 Table — Fully conserved sites throughout the nine domains are indicated with blue boxes, and partially conserved sites are indicated with red boxes. Three disulfide bonds are formed in each CR domain between 1–5 cysteines, 2–3 and 4–6 cysteines. (B) Disulfide bond distributions for various homology modeling templates. The templates with two disulfide bond pairs which close to the human P-selectin CR domain are highlighted with gray boxes. (DOCX) [file pone.0118083.s014.docx]

A

|  |
| --- |

B

| Templates  Disulfide bonds | 2G7I | 1RID | 1OK3 | 1GKN | 2RLQ | 1PPQ |
| --- | --- | --- | --- | --- | --- | --- |
| 1-5 | √ |  |  |  | √ |  |
| 2-3 |  |  |  |  |  |  |
| 4-6 | √ | √ | √ |  | √ |  |
